# Supplementary material for: Impact of sex on chemotherapy toxicity and efficacy in biliary tract cancer: Analysis of ABC, BILCAP trials and population data
Source: JHEP Rep. 2026 Feb 20;8(5):101777. doi: 10.1016/j.jhepr.2026.101777 (PMC13081176; doi:10.1016/j.jhepr.2026.101777)
Supplement: Multimedia component 1 [file mmc1.pdf]

Impact of sex on chemotherapy toxicity and efficacy in biliary tract cancer: Analysis of ABC, BILCAP trials and population data

Anna D. Wagner, Andre Lopes, Pinkie Chambers, Juan W. Valle, John Primrose, Chris Twelves, Luke Steventon, Zhe Wang, David Dodwell, John Bridgewater

Table of contents

Expanded Methodology – Population Study……………………………………………...…2

Fig. S1……………………………………………………………………………………...…3

Fig. S2……………………………………………………………………………………...…5

Fig. S3……………………………………………………………………………………...…6

Fig. S4……………………………………………………………………………………...…7

Fig. S5……………………………………………………………………………………...…8

Fig. S6……………………………………………………………………………………...…9

Fig. S7…………………………………………………………………………………….....10

Table S1……………………………………………………………………………………..11

Table S2……………………………………………………………………………………..12

Table S3……………………………………………………………………………………..14

Expanded Methodology - Population Study

Methods

The National Cancer Registry Database (NCRD) records diagnoses of all cancer patients treated within the NHS or NHS-funded centres in England. This registry provides a comprehensive record of >99% of patients with cancer in England. Cancer diagnosis is recorded using standardised ICD-10 codes. The Systemic Anti-Cancer Therapy (SACT) Dataset provided systemic treatment information that was linked to the NCRD patient records.

Inclusion and exclusion criteria

Patients with a diagnosis of biliary tract cancers were identified using ICD-10 codes (BTC, C22, extrahepatic cancer, C23, gallbladder cancer, C24, extrahepatic bile duct cancer).

Patients receiving cisplatin + gemcitabine combination or capecitabine monotherapy as first-line therapy were identified algorithmically using drug administration data available in the Systemic Anti-Cancer Therapy (SACT) Dataset.

Statistical analysis

Kaplan-Meier estimator was used to compare 1 and 5 year all-cause mortality between male and female treatment groups for each SACT protocol.

Cox regression analysis was performed for BTC patients treated with cisplatin + gemcitabine, or capecitabine monotherapy respectively as first-line systemic therapy. 1 year and 5 year all-cause mortality time was calculated as time from first chemotherapy treatment to death by any cause.

Results:

7,196 patients with a diagnosis of BTC were identified in the NCRD registry. Of these, 5,038 patients had a corresponding record of systemic treatment in the SACT dataset.

Of 5,565 patients who received SACT for BTC, 3,362 received cisplatin + gemcitabine in the first-line setting and 591 received capecitabine monotherapy.

Median age was 66 (IQR 58-72) overall. The study period was 02/04/2012 (first patient initiating chemotherapy) to 16/06/2022 (last patient initiating chemotherapy).

Fig. S1A: Patient disposition across trials and analysis subgroups

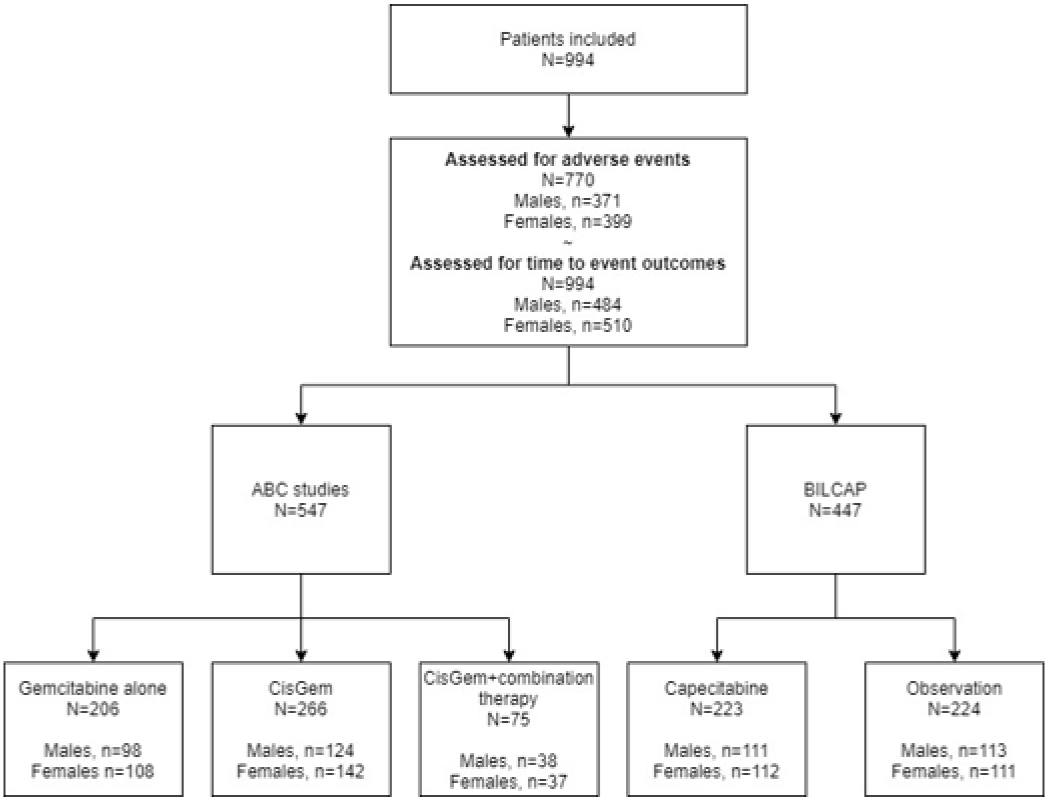

Fig. S1B: Patients included in population analysis

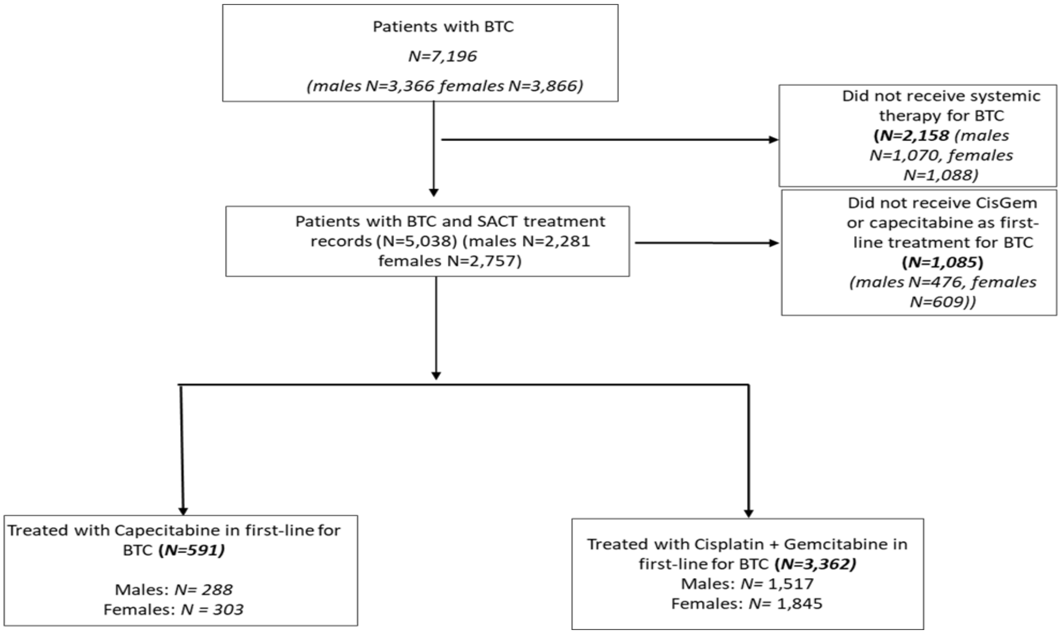

Abbreviations: BTC- biliary tract cancers; SACT- systemic anticancer treatments

Fig. S2: Grade 3-5 adverse events reported in ABC studies in ≥15 patients.

	Males N(%), N=260	Females N(%), N=287	OR(95%CI)	p
General disorders and administration site conditions				
Fatigue	10 (3.8%)	23 (8.0%)	2.18 (1.02;4.67)	0.045
Pain	24 (9.2%)	32 (11.1%)	1.23 (0.71;2.16)	0.460
Gastrointestinal disorders				
Nausea	7 (2.7%)	15 (5.2%)	1.99 (0.80;4.97)	0.139
Vomiting	13 (5.0%)	27 (9.4%)	1.97 (1.00;3.91)	0.052
Ascites	6 (2.3%)	9 (3.1%)	1.37 (0.48;3.90)	0.555
Abdominal pain	8 (3.1%)	11 (3.8%)	1.26 (0.50;3.17)	0.630
Diarrhea	12 (4.6%)	14 (4.9%)	1.06 (0.48;2.34)	0.885
Blood and lymphatic system disorders				
Febrile neutropenia	16 (6.2%)	26 (9.1%)	1.52 (0.80;2.90)	0.205
Anemia	22 (8.5%)	21 (7.3%)	0.85 (0.46;1.59)	0.620
Nervous system disorders				
Lethargy	40 (15.4%)	58 (20.2%)	1.39 (0.89;2.17)	0.143
Investigations				
Aspartate aminotransferase increased	18 (6.9%)	26 (9.1%)	1.34 (0.72;2.50)	0.360
Blood bilirubin increased	28 (10.8%)	35 (12.2%)	1.15 (0.68;1.95)	0.602
Alanine aminotransferase increased	29 (11.2%)	32 (11.1%)	1.00 (0.59;1.70)	0.999
GGT increased	40 (15.4%)	40 (13.9%)	0.89 (0.55;1.43)	0.632
Neutrophil count decreased	70 (26.9%)	68 (23.7%)	0.84 (0.57;1.24)	0.385
Alkaline phosphatase increased	10 (3.8%)	9 (3.1%)	0.81 (0.32;2.02)	0.651
Platelet count decreased	27 (10.4%)	23 (8.0%)	0.75 (0.42;1.35)	0.338
White blood cell decreased	42 (16.2%)	32 (11.1%)	0.65 (0.40;1.07)	0.089
Respiratory, thoracic and mediastinal disorders				
Dyspnea	9 (3.5%)	12 (4.2%)	1.22 (0.50;2.94)	0.662
Vascular disorders				
Thromboembolic event	17 (6.5%)	17 (5.9%)	0.90 (0.45;1.80)	0.766
Hypertension	23 (8.8%)	15 (5.2%)	0.57 (0.29;1.11)	0.100
Metabolism and nutrition disorders				
Anorexia	12 (4.6%)	11 (3.8%)	0.82 (0.36;1.90)	0.649
Hypernatremia/Hyponatremia	8 (3.1%)	7 (2.4%)	0.79 (0.28;2.20)	0.649
Infections and infestations				
Sepsis	9 (3.5%)	8 (2.8%)	0.80 (0.30;2.10)	0.651
Biliary tract infection	23 (8.8%)	18 (6.3%)	0.69 (0.36;1.31)	0.256
Infections and infestations (unspecified)	29 (11.2%)	21 (7.3%)	0.63 (0.35;1.13)	0.122

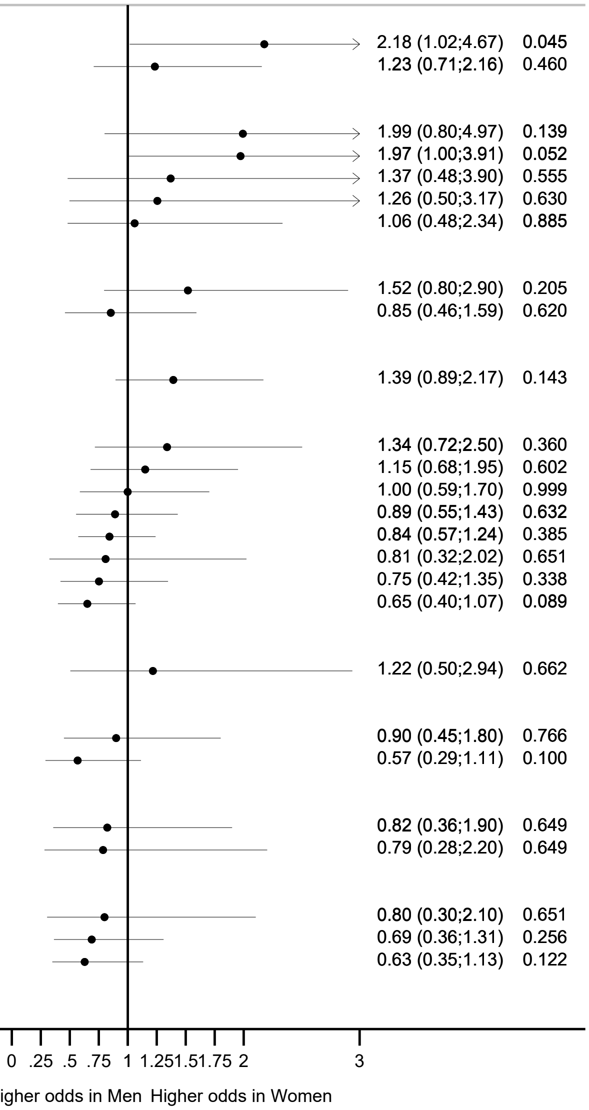

Fig. S2 depicts grade 3-5 AEs in the ABC studies reported in ≥15 patients. None of these AEs achieved a significance level of 0.01, although this was approached by fatigue (23, 8.0% vs 10, 3.8%; OR: 2.18, 95%CI: 1.02; 4.67, p=0.045) significance. Other relevant reported AEs grade ≥3 were numerically more frequent in females (58, 20.2% vs 40, 15.4%; OR: 1.39, 95%CI: 0.89; 2.17, p=0.14), vomiting (27, 9.4% vs 13, 5.0%; OR:

1.97, 95%CI: 1.00; 3.91, p=0.05), and nausea (15, 5.2% vs 7, 2.7%; OR: 1.99, 95%CI: 0.80; 4.97, p=0.14). In contrast, trends were reported towards greater rates in males of grade ≥3 leukopenia (42, 16.2% vs 32, 11.1%; OR: 0.65, 95%CI: 0.40; 1.07, p=0.09), hypertension (23, 8.8% vs 15, 5.2%; OR: 0.57, 95%CI: 0.29; 1.11, p=0.10) and infections/infestations (29, 11.2% vs 21, 7.3%; OR: 0.63, 95%CI: 0.35; 1.13, p=0.12).

Fig. S3. Adverse event of any grade (including grade 5) reported in BILCAP in ≥15 patients

CTCAE term 4.03	Males N(%), N=111	Females N(%), N=112	OR(95%CI)	p
Investigations				
Weight loss	11 (9.9%)	18 (16.1%)	1.74 (0.78;3.88)	0.175
Neutrophil count decreased	26 (23.4%)	23 (20.5%)	0.84 (0.45;1.59)	0.603
Platelet count decreased	14 (12.6%)	12 (10.7%)	0.83 (0.37;1.89)	0.659
Investigations (unspecified)	23 (20.7%)	19 (17.0%)	0.78 (0.40;1.53)	0.474
Alkaline phosphatase increased	10 (9.0%)	8 (7.1%)	0.78 (0.29;2.05)	0.610
Blood bilirubin increased	27 (24.3%)	18 (16.1%)	0.60 (0.31;1.16)	0.127
Blood and lymphatic system disorders				
Anemia	47 (42.3%)	37 (33.0%)	0.67 (0.39;1.16)	0.152
Eye disorders				
Eye disorders (unspecified)	13 (11.7%)	5 (4.5%)	0.35 (0.12;1.02)	0.055
Gastrointestinal disorders				
Vomiting	16 (14.4%)	34 (30.4%)	2.59 (1.33;5.03)	0.005
Nausea	42 (37.8%)	66 (58.9%)	2.36 (1.38;4.03)	0.002
Diarrhea	62 (55.9%)	75 (67.0%)	1.60 (0.93;2.76)	0.089
Mucositis oral	45 (40.5%)	56 (50.0%)	1.47 (0.86;2.49)	0.157
Abdominal pain	31 (27.9%)	40 (35.7%)	1.43 (0.81;2.53)	0.213
Constipation	17 (15.3%)	18 (16.1%)	1.06 (0.51;2.18)	0.877
Dyspepsia	11 (9.9%)	8 (7.1%)	0.70 (0.27;1.81)	0.461
General disorders and administration site conditions				
Fatigue	80 (72.1%)	95 (84.8%)	2.17 (1.12;4.20)	0.022
Fever	18 (16.2%)	15 (13.4%)	0.80 (0.38;1.68)	0.553
Injury, poisoning and procedural complications				
Wound complication	5 (4.5%)	10 (8.9%)	2.08 (0.69;6.29)	0.195
Metabolism and nutrition disorders				
Anorexia	11 (9.9%)	17 (15.2%)	1.63 (0.72;3.65)	0.238
Musculoskeletal and connective tissue disorders				
Arthralgia	6 (5.4%)	13 (11.6%)	2.30 (0.84;6.28)	0.105
Back pain	7 (6.3%)	8 (7.1%)	1.14 (0.40;3.27)	0.803
Nervous system disorders				
Dizziness	6 (5.4%)	14 (12.5%)	2.50 (0.92;6.76)	0.071
Dysgeusia	12 (10.8%)	14 (12.5%)	1.18 (0.52;2.68)	0.695
Nervous system disorders (unspecified)	10 (9.0%)	10 (8.9%)	0.99 (0.40;2.48)	0.983
Skin and subcutaneous tissue disorders				
Alopecia	4 (3.6%)	16 (14.3%)	4.46 (1.44;13.80)	0.010
Nail discoloration	4 (3.6%)	11 (9.8%)	2.91 (0.90;9.45)	0.075
Skin and subcutaneous tissue disorders (unspecified)	84 (75.7%)	91 (81.3%)	1.39 (0.73;2.65)	0.312
Pruritus	12 (10.8%)	7 (6.3%)	0.55 (0.21;1.45)	0.228

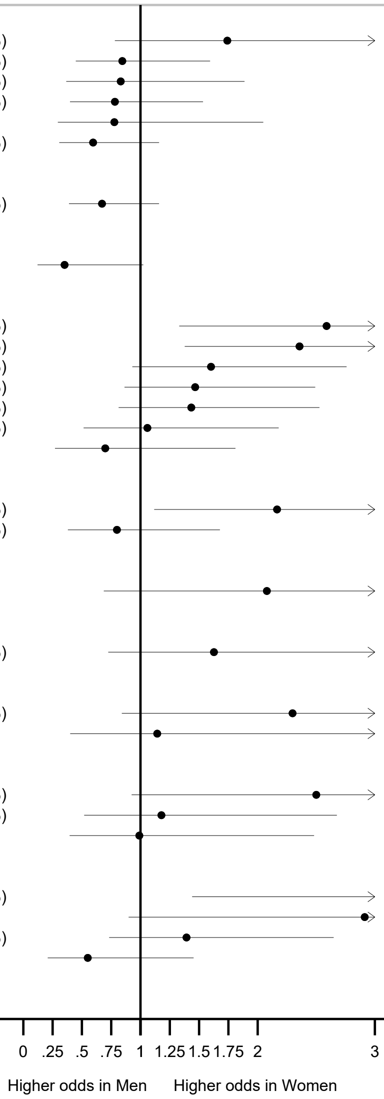

Fig. S3. Laboratory AEs again appeared more common in males, but differences were not statistically significant. Most clinical AEs were numerically greater in females; however, only vomiting (34, 30.4% vs 16, 14.4%, $p \leq 0.01$), nausea (66, 58.9% vs 42, 37.8%, $p \leq 0.01$), and alopecia (16, 14.3% vs 4, 3.6%, p=0.01) achieved statistical significance, while fatigue approached statistical significance (95, 84.8% vs 80, 72.1%, p=0.02).

Fig. S4. Overall survival by sex in BILCAP

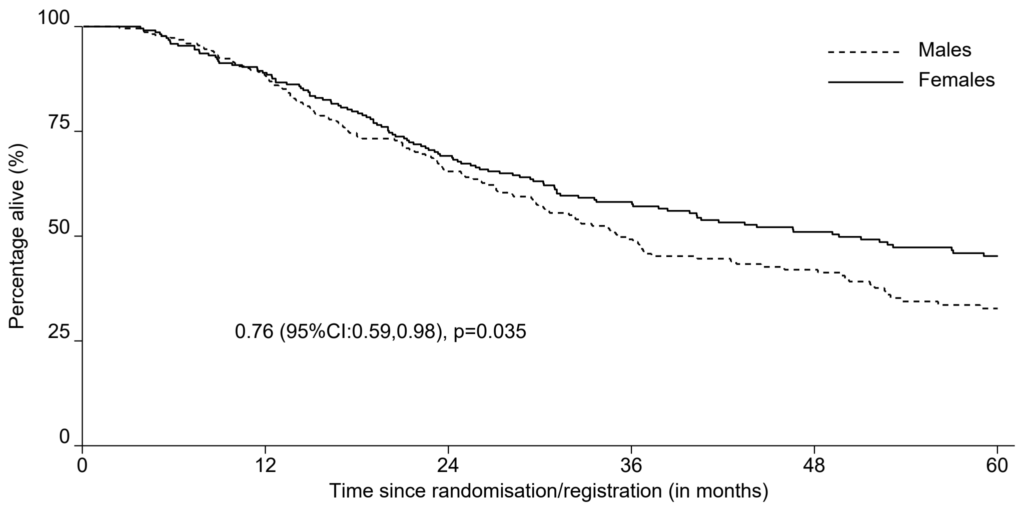

Number at risk

Males	224	(26)	195	(50)	142	(33)	89	(12)	61	(12)	0
Females	223	(24)	193	(43)	150	(23)	111	(13)	89	(9)	0

Fig. S5: 5-year OS by sex in metastatic BTC patients treated with first-line Cisplatin + Gemcitabine as per ABC protocol (population study).

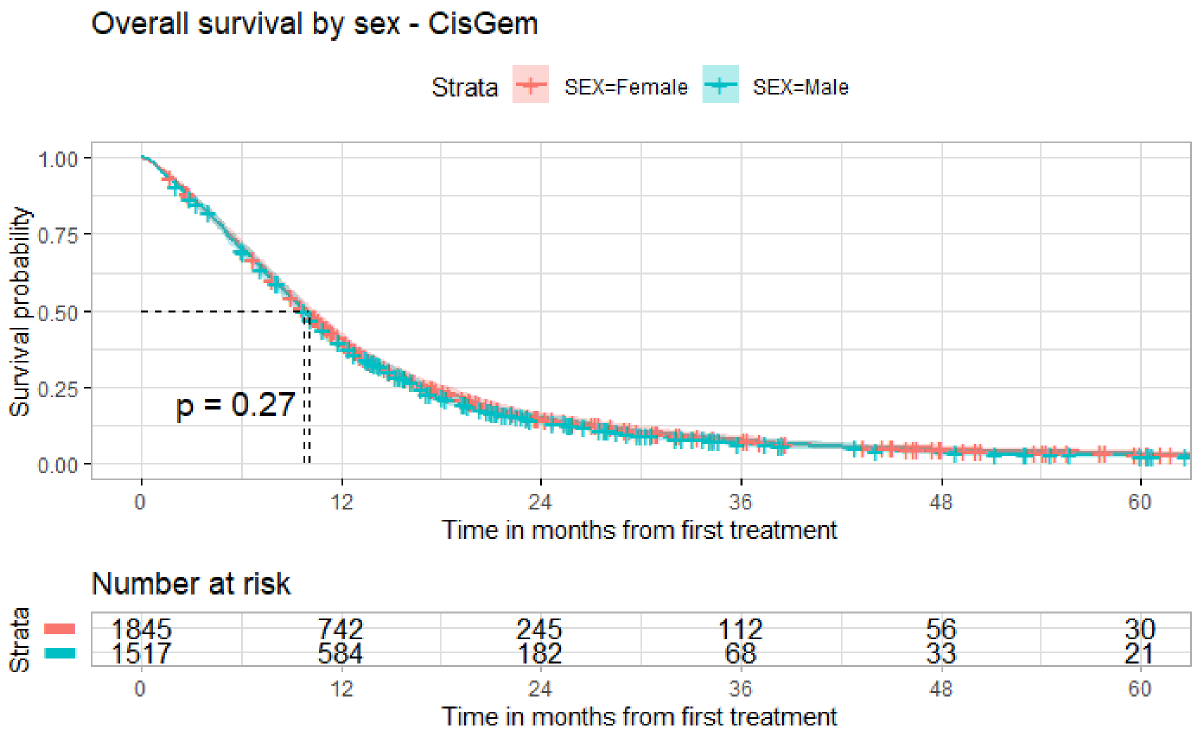

Fig. S6: 5-year OS by sex in patients with localized/ locally advanced BTC patients treated with adjuvant Capecitabine (population study).

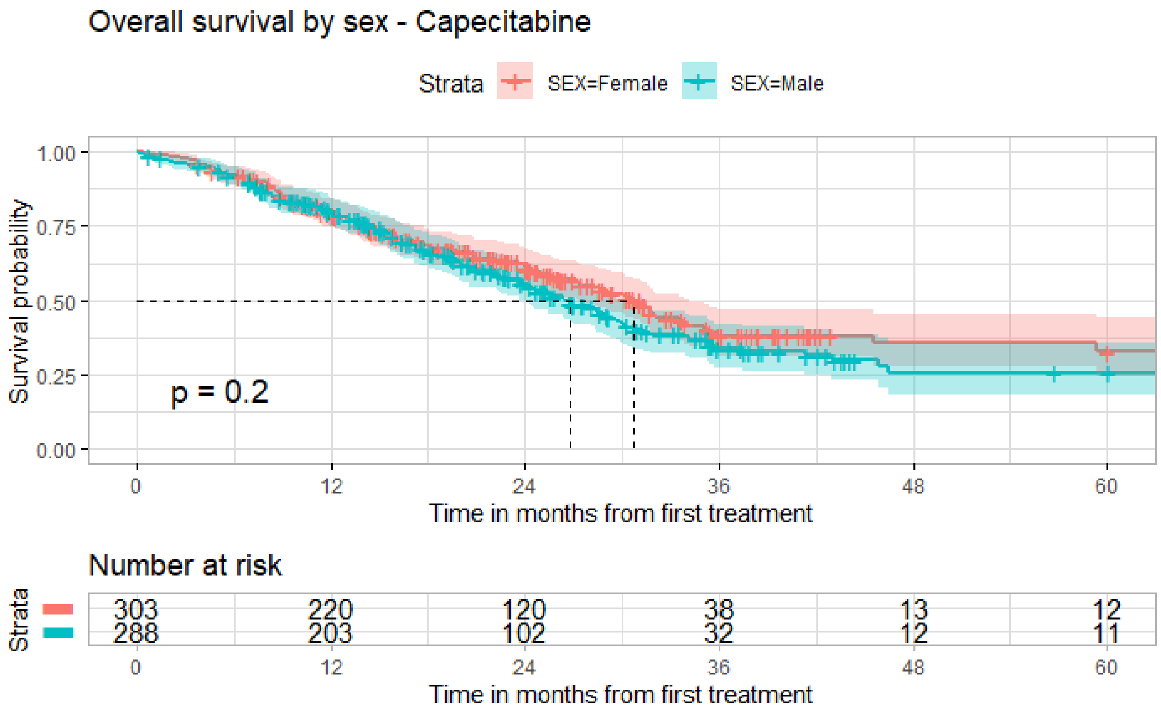

Fig. S7: 5-year OS by sex in gallbladder cancer patients treated with Capecitabine as per BILCAP protocol (population study).

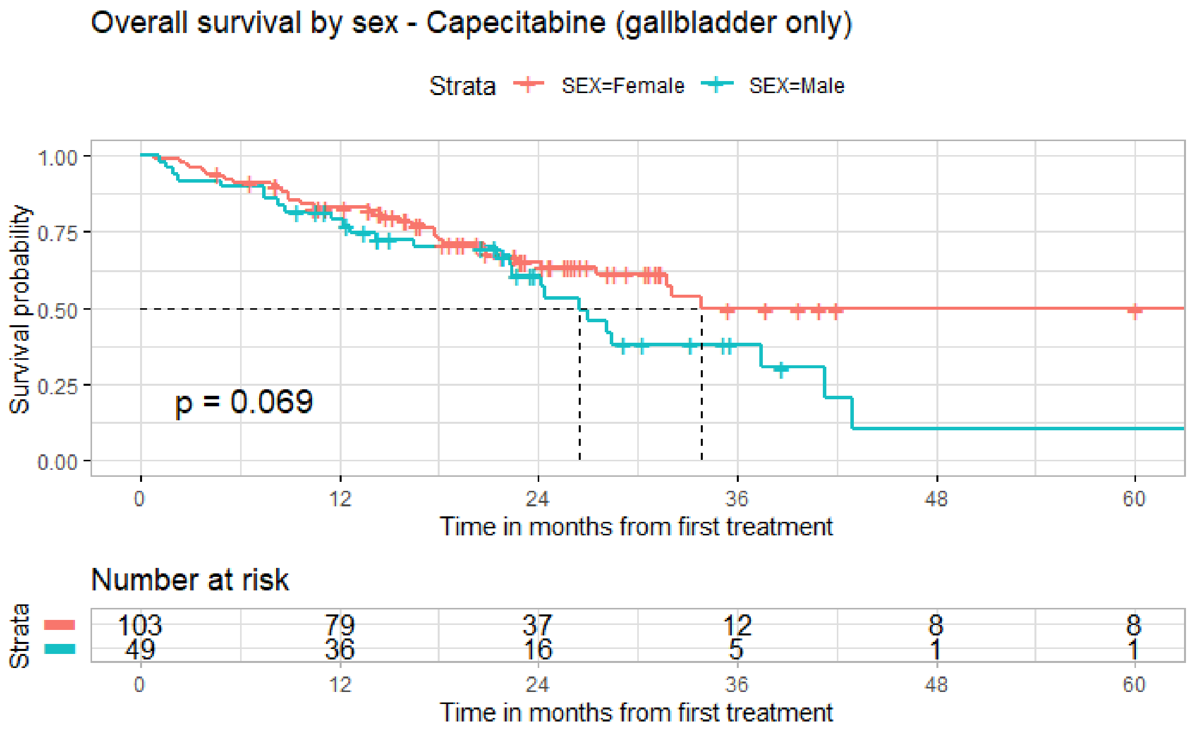

Table S1: Descriptive statistics of patients in national data for England treated with cisplatin + gemcitabine or capecitabine monotherapy

Characteristic	Overall N = 3,953[1]	Cisplatin + Gemcitabine N = 3,362[1]	Capecitabine N = 591[1]
Sex			
Female	2,148 (54%)	1,845 (55%)	303 (51%)
Male	1,805 (46%)	1,517 (45%)	288 (49%)
Age	66 (58, 72)	66 (58, 71)	67 (59, 73)
Diagnosis (ICD-10 code)			
Intrahepatic cholangiocarcinoma (C22.1)	2,120 (53%)	1,883 (56%)	237 (40%)
Cancer of gallbladder (C23.0)	1,059 (27%)	906 (27%)	153 (26%)
Cancer of extrahepatic or unspecified/other cancer of biliary tract (C24.0, C24.8, C24.9)	774 (19%)	573 (17%)	201 (34%)
Stage			
1	127 (3.2%)	72 (2.1%)	55 (9.3%)
2	466 (12%)	275 (8.2%)	191 (32%)
3	499 (13%)	350 (10%)	149 (25%)
4	1,774 (45%)	1,691 (50%)	83 (14%)
Unknown	1,087 (27%)	974 (29%)	113 (19%)
Ethnicity			
Asian	192 (4.9%)	166 (4.9%)	26 (4.4%)
Black	102 (2.6%)	94 (2.8%)	8 (1.4%)
Mixed Race	21 (0.5%)	18 (0.5%)	3 (0.5%)
Other	76 (1.9%)	64 (1.9%)	12 (2.0%)

Characteristic	Overall N = 3,953[1]	Cisplatin + Gemcitabine N = 3,362[1]	Capecitabine N = 591[1]
Unknown	95 (2.3%)	81 (2.3%)	14 (2.4%)
White	3,467 (88%)	2,939 (88%)	528 (89%)

[1] n (%); Median (IQR)

Table S2: Male vs female survival in real-world patients receiving cisplatin + gemcitabine.

Characteristic	HR[1]	95% CI[1]	p-value
Sex			
Female	0.97	0.88, 1.14	0.6
Male	-	-	-
Age	1.01	1.00, 1.01	0.001
Charlson comorbidity index			
0	0.86	0.76, 0.97	0.018
≥1	—	—	
Ethnicity			
Asian	—	—	
Black	1.36	0.93, 1.99	0.11
Mixed Race	1.22	0.61, 2.45	0.6
Other	1.37	0.91, 2.06	0.13
Unknown	2.01	1.40, 2.88	<0.001

**Characteristic**	**HR**[1]	**95% CI**[1]	**p-value**
White	1.44	1.13, 1.84	0.003
Stage			
1	—	—	
2	1.20	0.78, 1.86	0.4
3	1.26	0.82, 1.92	0.3
4	2.16	1.45, 3.21	<0.001
Unknown	1.58	1.06, 2.37	0.026

[1] HR = Hazard Ratio, CI = Confidence Interval

Table S3: Cox regression analysis of relative risk of male vs female sex in real-world patients receiving single-agent capecitabine.

Characteristic	HR[1]	95% CI[1]	p-value
Sex			
Female	0.85	0.57, 1.26	0.4
Male	-	-	-
Age	1.01	1.0, 1.03	0.2
Ethnicity			
White	—	—	
Asian	0.89	0.28, 2.83	0.8
Black	1.27	0.17, 9.40	0.8
Mixed Race	0.00	0.00, Inf	>0.9
Other	3.08	1.10, 8.60	0.032
Unknown	3.27	1.30, 8.24	0.012
Charlson comorbidity index			
0	—	—	
≥1	1.29	0.80, 2.08	0.3
Stage			
1	—	—	
2	1.00	0.32, 3.15	>0.9
3	2.99	1.04, 8.60	0.043
4	9.93	3.49, 28.3	<0.001
Unknown	4.04	1.38, 11.8	0.011

[1] HR = Hazard Ratio, CI = Confidence Interval
