# Supplementary material for: Impact of sex on chemotherapy toxicity and efficacy in biliary tract cancer: Analysis of ABC, BILCAP trials and population data
Source: JHEP Rep. 2026 Feb 20;8(5):101777. doi: 10.1016/j.jhepr.2026.101777 (PMC13081176; doi:10.1016/j.jhepr.2026.101777)
Supplement: Multimedia component 2 [file mmc2.docx]

**Journal of Hepatology**

**CTAT methods**

Tables for a “Complete, Transparent, Accurate and Timely account” (CTAT) are now mandatory for all revised submissions. The aim is to enhance the reproducibility of methods.

- Only include the parts relevant to your study
- Refer to the CTAT in the main text as ‘Supplementary CTAT Table’
- Do not add subheadings
- Add as many rows as needed to include all information
- Only include one item per row

**If the CTAT form is not relevant to your study, please outline the reasons why:**

|  |
| --- |

- 1. **Antibodies**

| **Name** | **Citation** | **Supplier** | **Cat no.** | **Clone no.** |
| --- | --- | --- | --- | --- |
| **N/A** |  |  |  |  |

- 1. **Cell lines**

| **Name** | **Citation** | **Supplier** | **Cat no.** | **Passage no.** | **Authentication test method** |
| --- | --- | --- | --- | --- | --- |
| **N/A** |  |  |  |  |  |

- 1. **Organisms**

| **Name** | **Citation** | **Supplier** | **Strain** | **Sex** | **Age** | **Overall n number** |
| --- | --- | --- | --- | --- | --- | --- |
| **N/A** |  |  |  |  |  |  |

- 1. **Sequence based reagents**

| **Name** | **Sequence** | **Supplier** |
| --- | --- | --- |
| **N/A** |  |  |

- 1. **Biological samples**

| **Description** | **Source** | **Identifier** |
| --- | --- | --- |
| **N/A** |  |  |

- 1. **Deposited data**

| **Name of repository** | **Identifier** | **Link** |
| --- | --- | --- |
| **N/A** |  |  |

- 1. **Software**

| **Software name** | **Manufacturer** | **Version** |
| --- | --- | --- |
| **STATA** | **StataCorp** | **V17** |
| **RStudio** | **Posit PBC** | **v4.3.0** |

- 1. **Other (e.g. drugs, proteins, vectors etc.)**

| **N/A** |  |  |
| --- | --- | --- |
|  |  |  |

- 1. **Please provide the details of the corresponding methods author for the manuscript:**

| Clinical trial analysis: Anna Wagner, Département d'oncologie, University of Lausanne, Switzerland, Dorothea.Wagner@chuv.ch  Population data analysis: Luke Steventon, School of Pharmacy, University College London, United Kingdom, luke.steventon.22@ucl.ac.uk |
| --- |

**2.0 Please confirm for randomised controlled trials all versions of the clinical protocol are included in the submission. These will be published online as supplementary information.**

| Trial protocols have previously been published for ABC02, ABC03, ABC04. |
| --- |
