# Supplementary material for: Impact of sex on chemotherapy toxicity and efficacy in biliary tract cancer: Analysis of ABC, BILCAP trials and population data
Source: JHEP Rep. 2026 Feb 20;8(5):101777. doi: 10.1016/j.jhepr.2026.101777 (PMC13081176; doi:10.1016/j.jhepr.2026.101777)
Supplement: Multimedia component 4 [file mmc4.pdf]

Research article

JHEP|Reports

Impact of sex on chemotherapy toxicity and efficacy in biliary tract cancer: Analysis of ABC, BILCAP trials and population data

Authors

Anna D. Wagner, Andre Lopes, Pinkie Chambers, …, Zhe Wang, David Dodwell, John Bridgewater

Correspondence

p.chambers@ucl.ac.uk (P. Chambers).

Graphical abstract

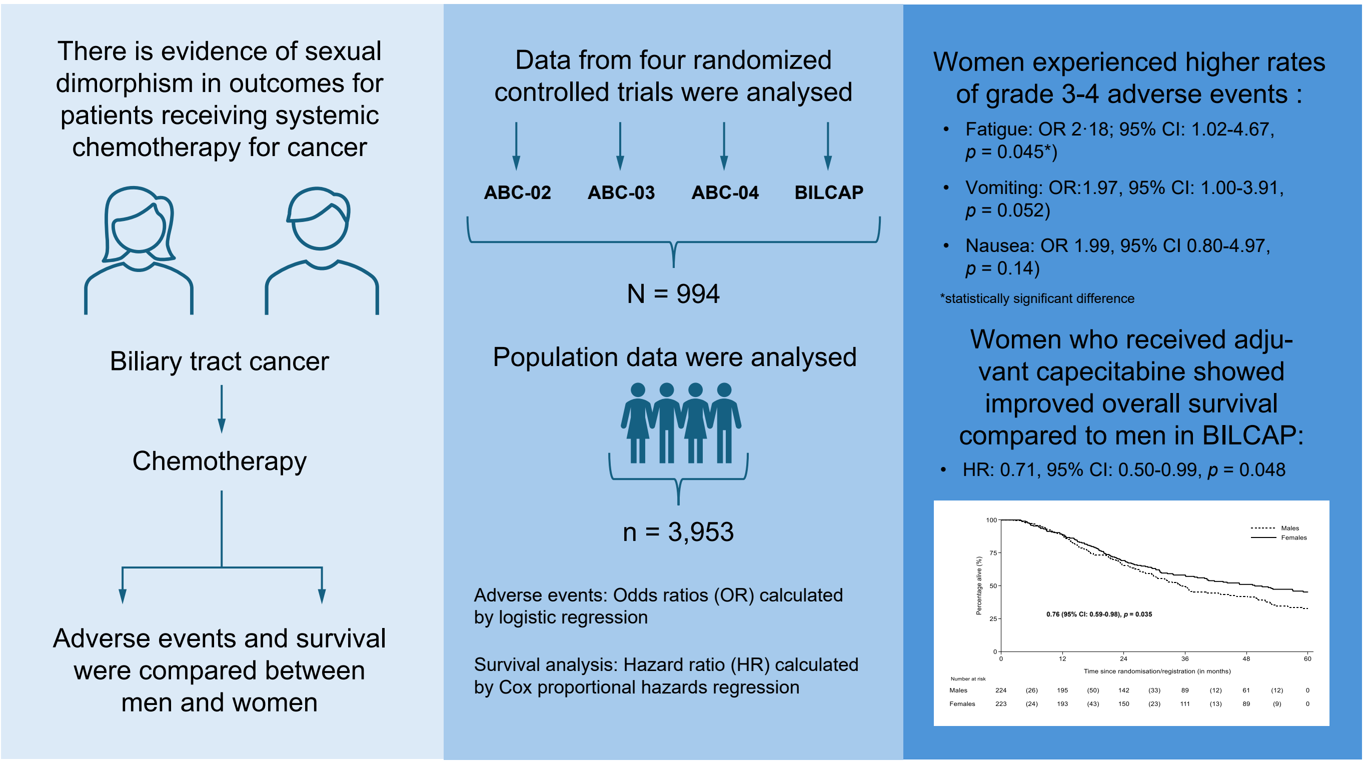

Highlights:

- There is evidence of sexual dimorphism in treatment outcomes for patients with cancer.
- Outcomes for patients receiving chemotherapy for biliary tract cancers were investigated in RCT and population cohorts.
- Females experienced higher rates of grade 3 and 4 adverse events: fatigue, lethargy and nausea and vomiting.
- Despite more adverse events, females experienced superior survival compared to males across all biliary tract cancers.
- This work underlines the need to consider sex differences in the design, analysis and interpretation of clinical trials.

Impact and implications:

This study investigated the impact of biological sex on treatment outcomes in patients receiving chemotherapy for biliary tract cancers, which are typically associated with poor outcomes. Analysis of ABC and BILCAP clinical trials found a higher incidence of severe grade adverse events in women receiving cisplatin/gemcitabine and adjuvant capecitabine, relative to males, whilst overall survival was superior in women in the BILCAP trial. These findings are important for clinicians treating patients with biliary tract cancers and should be considered in the design and analysis of future clinical trials in biliary tract cancer, as the role of biological sex may an important determinant of chemotherapy response.

https://doi.org/10.1016/j.jhepr.2026.101777
© 2026 The Authors. Published by Elsevier B.V. on behalf of European Association for the Study of the Liver (EASL). This is an open access article under the CC BY license (http://creativecommons.org/licenses/by/4.0/). JHEP Reports, 2026, 8, 1–9

Impact of sex on chemotherapy toxicity and efficacy in biliary tract cancer: Analysis of ABC, BILCAP trials and population data

Anna D. Wagner[1], Andre Lopes[2], Pinkie Chambers[3,*], Juan W. Valle[4], John Primrose[5], Chris Twelves[6], Luke Steventon[3], Zhe Wang[7], David Dodwell[7], John Bridgewater[8]

JHEP Reports **2026**. vol. 8 | 1–9

Check for updates

Background & Aims: Increasing evidence suggests sexual dimorphism in treatment effects across cancers; however, its impact in biliary tract cancer (BTC) remains unclear. We compared toxicity and efficacy of palliative and adjuvant chemotherapy between male and female patients with BTC.

Methods: We conducted a retrospective cohort analysis of individual patient data from four randomized controlled trials in BTC and English population-based data. Study outcomes were adverse events and overall survival (OS), compared by sex.

Results: Among 994 trial participants (49% male, 51% female) included in time-to-event analyses, 770 were evaluable for adverse events. Population data included 3,953 patients (46% male, 54% female) for OS analysis. Females experienced higher rates of grade 3/4 fatigue (odds ratio [OR] 2.18; 95% CI 1.02–4.67; p = 0.045). Higher rates of grade 3/4 vomiting (OR 1.97; 95% CI 1.00–3.91; p = 0.052), nausea (OR 1.99; 95% CI 0.80–4.97; p = 0.14), and fatigue in BILCAP (OR 2.31; 95% CI 0.77–6.88; p = 0.13) were observed in females but were not statistically significant. OS was similar between sexes in ABC trials (hazard ratio [HR] 0.94; 95% CI 0.79–1.11; p = 0.45) and in population data (HR 1.03; 95% CI 0.79–1.11; p = 0.45). In BILCAP, the HR for adjuvant capecitabine *vs*. observation was 0.71 in males (95% CI 0.50–1.00; p = 0.048) and 0.91 in females (95% CI 0.63–1.32; p = 0.625). Females with gallbladder cancer demonstrated improved OS compared with males in BILCAP (HR 0.48; 95% CI 0.24–0.98; p = 0.04).

Conclusion: Sex differences in toxicity were observed, with higher rates of grade 3/4 fatigue in females. Survival outcomes were broadly similar; however, females with gallbladder cancer receiving adjuvant capecitabine showed improved survival compared with males. Although population analyses were limited by sample size, these findings warrant consideration in the design and interpretation of future BTC trials.

© 2026 The Authors. Published by Elsevier B.V. on behalf of European Association for the Study of the Liver (EASL). This is an open access article under the CC BY license (http://creativecommons.org/licenses/by/4.0/).

Introduction

The importance of sex and gender as modifiers of health, disease and medicine is increasingly recognized.[1] Marked differences in incidence and survival between male and female patients have been described for the majority of non-sex-related cancers and in different populations, with mortality generally being higher in males.[2,3] Biliary tract cancers (BTC) are comprised of cholangiocarcinoma (intrahepatic [iCCA] and extrahepatic [eCCA]) and gallbladder adenocarcinoma (GBC). The incidence rates are distributed unevenly between the sexes, with a characteristically higher incidence of iCCA in females and eCCA in males. GBC[4] develops significantly more frequently in females[5–8] with a worldwide female:male incidence rate ratio of 2:1, although this varies greatly with geography.[9,10] The association between high parity and risk of GBC is highly suggestive of a role of female sex hormones in its aetiology.[10] Furthermore, differences in biology of cancers that are not sex-related arising in male and female patients, also referred to as sexual dimorphism in cancer, are supported by accumulating evidence.[11–14] For example, among gastrointestinal cancers, diffuse type gastric[15] and right-sided colon cancers[16,17] are significantly more common in females, while rectal cancers are more common in males.[18] Additionally, sex can affect drug metabolism,[19] with greater toxicity reported in females for various types of anticancer treatment[20–24] over the past 15 years.

For patients with BTC, where the standard of care systemic anticancer treatments are established by studies in early stage[25] and advanced disease,[26–28] the impact of the patients' sex on treatment toxicity and outcomes is unknown.

The current exploratory analysis aims to describe, according to the patients' sex: i) AEs and treatment administration/dose modification; ii) efficacy, in terms of progression-free (PFS) and overall survival (OS) for palliative chemotherapy in advanced/metastatic disease and relapse-free (RFS) and OS

* Corresponding author. Address: UCL School of Pharmacy, London WC1E 6DD, UK, Tel.: +44 203447 9093.
E-mail address: p.chambers@ucl.ac.uk (P. Chambers).
https://doi.org/10.1016/j.jhepr.2026.101777

after adjuvant treatment of early disease in a large dataset of prospective BTC clinical trial participants. To validate our findings from trial data, we investigated the second objective, OS, using population data obtained from England. Of note, while "gender" is defined by the World Health Organization as the socially constructed roles, behaviours, activities, and attributes that a given society considers appropriate for men and women, "sex" refers to the biological bases that underlie female or male anatomy and physiology. Our study is related to biological differences and therefore the term "sex", will be used.

Patients and methods

Procedures and study participants

Four prospective advanced BTC (ABC) clinical trials were included, ABC-02[26] (gemcitabine +/- cisplatin), -03[28] (cisplatin/gemcitabine +/- cediranib), -04[27] (cisplatin/gemcitabine +/- selumetinib) and the BILCAP[25] adjuvant trial in surgically resected BTC. Population data for England[29] were accessed for those aged 18 or over with a diagnosis of BTC identified using ICD-10 codes (C22.1, C23.0, C24.0/C24.8/C24.9), between April 1, 2012, and December 31, 2019. In the population data we included those receiving cisplatin + gemcitabine combination or capecitabine monotherapy as first-line and adjuvant therapy, respectively.

Data analysis

Trial datasets

AEs were classified according to National Cancer Institute's Common Toxicity Criteria v4.03. The incidence of AEs was extracted, and logistic regression used to investigate the associations with biological sex. Separate analyses of AEs were conducted for ABC trials and BILCAP, as well as for all graded AEs (Fig. S3), and AEs with a severity grade of ≥3 (Fig. S2). Secondary outcomes were OS, PFS and RFS. OS was measured from randomization to death or the last day of follow-up; PFS and RFS were measured to the time to progression (PFS), recurrence, or the last day of follow-up. Interaction tests between sex and treatment were used to examine whether the influence of sex on OS, PFS, and RFS varied by treatment.

Given the higher incidence of GBC in women and its association with reproductive factors,[10] separate analyses of OS, PFS and RFS were conducted in patients with GBC. Additionally, the percentage of patients experiencing at least one dose reduction and the median time on treatment were compared using chi-square test and quantile regression respectively.

Time to event outcomes were compared between the sexes using Kaplan Meier plots and Cox regression. All statistical tests were two-sided. A p value of <0.05 was considered a statistically significant difference in survival where the hazard ratio did not cross the null.

Population data

National Cancer Registry and Analysis Service (NCRAS) [30] and Systemic Anti-Cancer Therapy (SACT) datasets.[29] were used to study the English patient population. These datasets record specific information such as patient age, diagnosis by ICD-10 code, follow-up status, and specific SACT received for their cancer. NCRAS has recorded all cancer diagnoses since 1971 in England and the reporting of SACT data has been mandated since 2014 for NHS cancer-treating sites. There is a known reporting lag of up to 5 years for cancer diagnoses to be collated into the NCRAS registry, which have been discussed by authors of the Data Resource Profile for this registry. However, this was not considered a significant proportion of patients with BTC that would impact the analysis presented here. The study period was also selected to account for this.

The outcome measure was OS in this group. OS was measured from date of treatment initiation to death or censoring date. Kaplan-Meier analysis was used to compare 1- and 5-year all-cause mortality between male and female patients for each treatment regimen, separating the patients with GBC to replicate the analysis of the trial data.

Multivariable Cox regression analysis was performed separately for patients receiving palliative cisplatin–gemcitabine for BTC and for those receiving adjuvant capecitabine monotherapy. One-year and 5-year survival was calculated as time from first chemotherapy treatment to death by any cause.

Trial data were analysed using STATA 17 and RStudio version 4·3·0 was used for population data analysis. Ethical approval for use of these data was granted under the Cancer Research UK Grant "Benefits and Risks of Cancer Treatments", favourable opinion given 10/06/2019, REC reference[18]/NS/0057).

Results

Patient characteristics

A total of 994 patients from ABC-02, ABC-03, ABC-04, and BILCAP were evaluable for time-to-event analyses, including AEs, comprising 484 (49%) males and 510 (51%) females (n = 410, 124, 13, and 447 per trial, respectively). Patients randomised to the control/observation group in the BILCAP study (n = 224) were not assessed for safety. Therefore, 770/994 (77%) patients were included in the analysis of AEs. All patients included in the ABC studies were analysed for efficacy, including those who received CisGem + experimental treatment. We considered this a valid approach as CisGem was given as the backbone treatment and no significant difference in survival was observed between treatment groups in the ABC-03/04 studies. The patient disposition across trials and analysis subgroups is depicted in Fig. S1A. Baseline patient and tumour characteristics are presented in Table 1.

Within the population data, a total of 3,362 patients receiving cisplatin+ gemcitabine and 591 patients receiving capecitabine were included from 14/11/2013, with the final treatment given on 16/06/2022. The included cohort is presented in Fig. S1B and characteristics of this cohort are provided in Table 1.

Tolerability

Of the 770 evaluable patients, 540 in the ABC trials and 230 in BILCAP, 371 (48%) were male and 399 (52%) were female. Overall, the largest differences in grade 3/4 AEs were observed for fatigue, which was significantly more common in females

Table 1. Baseline characteristics of the ABC, BILCAP and population data cohorts.

	All		ABC-studies		BILCAP		Population data		
	Males	Females	Males	Females	Males	Females	All	Males	Females
**Baseline characteristics**	n = 484	n = 510	n = 260	n = 287	n = 224	n = 223	n = 3,953	n = 1,805	n = 2,148
Primary site									
iCCA	100 (21)	111 (22)	65 (25)	62 (22)	35 (16)	49 (22)	2,120 (54)	1,059 (59)	1,061 (49)
hCCA	119 (25)	78 (15)	39 (15)	30 (10)	80 (36)	48 (22)	0 (0)	0 (0)	0 (0)
eCCA	68 (14)	62 (12)	68 (26)	62 (22)	0 (0)	0 (0)	772 (20)	433 (24)	339 (16)
Gallbladder	72 (15)	171 (34)	53 (20)	111 (39)	19 (8)	60 (27)	1,059 (27)	312 (17)	747 (35)
Ampulla	23 (5)	8 (2)	23 (9)	8 (3)	0 (0)	0 (0)	0 (0)	0 (0)	0 (0)
CCA – NS*	100 (21)	76 (15)	10 (4)	10 (3)	90 (40)	66 (30)	2 (0)	1 (0)	1 (0)
Not reported	2 (<1)	4 (1)	2 (1)	4 (1)	0 (0)	0 (0)	0 (0)	0 (0)	0 (0)
ECOG perform status									
0	207 (43)	186 (36)	102 (39)	91 (32)	105 (47)	95 (43)	635 (16)	327 (18)	326 (15)
1	252 (52)	281 (55)	138 (53)	164 (57)	114 (51)	117 (52)	1,218 (31)	539 (30)	679 (32)
2	24 (5)	41 (8)	19 (7)	32 (11)	5 (2)	9 (4)	-	-	-
Not reported	1 (<1)	2 (<1)	1 (0)	0 (0)	0 (0)	2 (1)	2,082 (53)	939 (52)	1,143 (53)
Diagnosis grading									
Well differentiated	52 (11)	61 (12)	20 (8)	23 (8)	32 (14)	38 (17)	-	-	-
Moderately differentiated	204 (42)	196 (38)	88 (34)	82 (29)	116 (52)	114 (51)	-	-	-
Poorly differentiated	111 (23)	113 (22)	48 (18)	56 (20)	63 (28)	57 (26)	-	-	-
Not available	117 (24)	140 (27)	104 (40)	126 (44)	13 (6)	14 (6)	3,953 (100)	1,805 (100)	2,148 (100)
T-stage									
0	5 (1)	2 (0)	5 (2)	2 (1)	0 (0)	0 (0)	0 (0)	0 (0)	0 (0)
1	30 (6)	33 (6)	14 (5)	13 (5)	16 (7)	20 (9)	127 (3)	61 (3)	66 (3)
2	104 (21)	116 (23)	29 (11)	28 (10)	75 (33)	88 (39)	466 (12)	247 (14)	219 (10)
3	180 (37)	190 (37)	59 (23)	87 (30)	121 (54)	103 (46)	499 (13)	215 (12)	284 (13)
4	60 (12)	52 (10)	48 (18)	41 (14)	12 (5)	11 (5)	1,774 (45)	760 (42)	1,014 (47)
Not available	105 (22)	117 (23)	105 (40)	116 (40)	0 (0)	1 (0)	1,087 (27)	522 (29)	565 (26)
N-stage									
0	170 (35)	186 (36)	54 (21)	66 (23)	116 (52)	120 (54)	-	-	-
1	209 (43)	197 (39)	101 (39)	95 (33)	108 (48)	102 (46)	-	-	-
2	0 (0)	1 (<1)	0 (0)	1 (0)	0 (0)	0 (0)	-	-	-
Not available	105 (22)	126 (25)	105 (40)	125 (44)	0 (0)	1 (0)	3,953 (100)	1,805 (100)	2,148 (100)
M-stage									
0	291 (60)	292 (57)	67 (26)	71 (25)	224 (100)	221 (99)	-	-	-
1	193 (40)	215 (42)	193 (74)	214 (75)	0 (0)	1 (0)	-	-	-
Not available	0 (0)	3 (1)	0 (0)	2 (1)	0 (0)	1 (0)	3,953 (100)	1,805 (100)	2,148 (100)
Prior therapy at entry									
No	57 (12)	85 (17)	57 (22)	85 (30)	0 (0)	0 (0)	-	-	-
Yes	203 (42)	202 (40)	203 (78)	202 (70)	0 (0)	0 (0)	-	-	-
Not reported	224 (46)	223 (44)	0 (0)	0 (0)	224 (100)	223 (100)	3,953 (100)	1,805 (100)	2,148 (100)
BMI									
Normal	228 (47)	215 (42)	128 (49)	127 (44)	100 (45)	88 (39)	1,210 (31)	554 (31)	656 (31)
Underweight	11 (2)	27 (5)	9 (3)	14 (5)	2 (1)	13 (6)	99 (3)	37 (2)	62 (3)
Overweight	175 (36)	149 (29)	87 (33)	76 (26)	88 (39)	73 (33)	1,033 (26)	507 (28)	526 (25)
Obese	56 (12)	109 (21)	28 (11)	66 (23)	28 (13)	43 (19)	621 (16)	267 (15)	354 (16)
Not available	14 (3)	10 (2)	8 (3)	4 (1)	6 (3)	6 (3)	990 (25)	440 (24)	550 (25)
Treatment									
Gem alone	98 (20)	108 (21)	98 (38)	108 (38)	0 (0)	0 (0)	0 (0)	0 (0)	0 (0)
CisGem	124 (26)	142 (28)	124 (48)	142 (49)	0 (0)	0 (0)	3,362 (85)	1,517 (84)	1,845 (86)
CisGem + cediranib	34 (7)	28 (5)	34 (13)	28 (10)	0 (0)	0 (0)	0 (0)	0 (0)	0 (0)
CisGem + selumetinib	4 (1)	9 (2)	4 (2)	9 (3)	0 (0)	0 (0)	0 (0)	0 (0)	0 (0)
Observation	113 (23)	111 (22)	0 (0)	0 (0)	113 (50)	111 (50)	0 (0)	0 (0)	0 (0)
Capecitabine	111 (23)	112 (22)	0 (0)	0 (0)	111 (50)	112 (50)	591 (15)	288 (16)	303 (14)

Data are presented as n (%).
eCCA, extrahepatic cholangiocarcinoma; hCCA, hilar cholangiocarcinoma; iCCA, intrahepatic cholangiocarcinoma.
*NS: not specified.

(odds ratio [OR] 2.18; 95% CI 1.02–4.67; p = 0.045). Higher rates of lethargy (OR 1.39; 95% CI 0.89–2.17; p = 0.14), vomiting (OR 1.97; 95% CI 1.00–3.91; p = 0.052), and nausea (OR 1.99; 95% CI 0.80–4.97; p = 0.14) were also observed in females with advanced BTC, although these were not statistically significant. In BILCAP, grade 3/4 fatigue was more frequent in females, but this difference was not significant (OR 2.31; 95% CI 0.77–6.88; p = 0.13).

ABC studies

All grade AEs reported in ≥15 patients are shown in Fig. 1. Laboratory adverse events were more frequent in males, with significantly higher rates of thrombocytopaenia (146, 56.2% *vs.* 125, 43.6%; p <0.01), elevated creatinine (28, 10.8% *vs.* 17, 5.9%; p = 0.04), weight loss (22, 8.5% *vs.* 11, 3.8%; p = 0.03), and hyperglycaemia (13, 5.0% *vs.* 5, 1.7%; p = 0.04). Anaemia

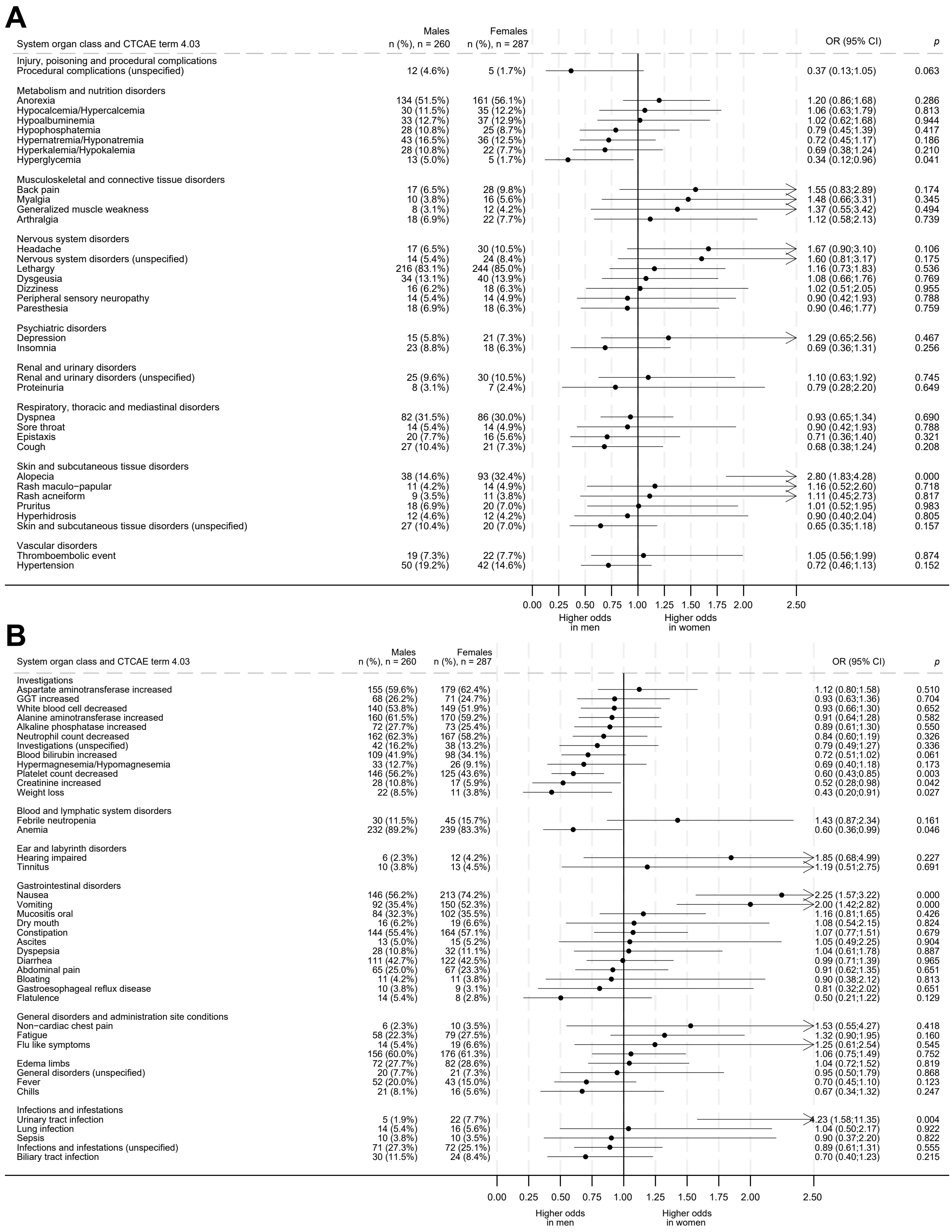

Fig. 1. Summary of adverse events for ABC studies. OR, odds ratio.

was also more common in males but did not reach statistical significance (232, 89.2% *vs.* 239, 83.3%; $p = 0.054$). In contrast, females had higher rates of clinical adverse events, including nausea (213, 74.2% *vs.* 146, 56.2%; $p < 0.01$), vomiting (150, 52.3% *vs.* 92, 35.4%; $p < 0.01$), urinary tract infection (22, 7.7% *vs.* 5, 1.9%; $p < 0.01$), and alopecia (93, 32.4% *vs.* 38, 14.6%; $p < 0.01$).

BILCAP study

All grade AEs are shown in Fig. S3.

Only fatigue, diarrhoea and skin disorders were reported as grade 3-5 AEs in ≥15 patients, with no differences between sexes reaching statistical significance for these events.

Efficacy

ABC studies

Median OS was 10.6 months (95% CI 9.1–11.9) for females and 10.2 months (95% CI 8.4–11.9) for males (HR 0.94; 95% CI 0.79–1.11; $p = 0.45$; Fig. 2). Median PFS was 6.5 months (95% CI 5.9–7.6) for females and 6.4 months (95% CI 5.8–7.1) for males (HR 1.03; 95% CI 0.86–1.22; $p = 0.77$). OS and PFS did not differ significantly by sex (test for interaction $p = 0.67$ and $p = 0.85$, respectively). Subgroup analysis of patients with GBC also showed no sex differences in OS ($p = 0.93$) or PFS ($p = 0.54$).

BILCAP study

As previously reported, adjuvant capecitabine improved OS compared with observation in the intention-to-treat analysis (median OS 51.1 months [95% CI 34.6–59.1] *vs.* 36.4 months [95% CI 29.7–44.5]), although this difference did not reach statistical significance (HR 0.81; 95% CI 0.63–1.04; $p = 0.10$).

Across the two arms of BILCAP, median OS was 49.6 months (95% CI not calculated) in females *vs.* 35.1 months (95% CI 30.3–44.5) in males (HR 0.76; 95% CI 0.59–0.98; $p = 0.04$; Fig. S4). There was no evidence of a sex difference in recurrence-free survival (RFS), with median RFS of 19.4 months in females and 20.8 months in males (HR 0.89; 95% CI 0.71–1.13; $p = 0.34$). The superior OS in females was observed in both the capecitabine and observation arms. In the capecitabine arm, median OS was 57.0 months (95% CI not calculated) in females *vs.* 42.9 months (95% CI 32.2–58.9) in males. In the observation arm, median OS was 44.2 months (95% CI not calculated) in females *vs.* 31.9 months (95% CI 23.9–36.9) in males. The interaction between sex and treatment did not reach statistical significance (interaction $p = 0.36$); however, the estimated treatment effect on OS was more pronounced in males (HR 0.71; 95% CI 0.50–1.00; $p = 0.05$) than in females (HR 0.91; 95% CI 0.63–1.32; $p = 0.63$) (Fig. 3A,B). The interaction between sex and treatment for RFS was similar to that observed for OS (interaction $p = 0.50$; Fig. 4A,B). Among patients with GBC in BILCAP, females had superior OS (HR 0.48; 95% CI 0.24–0.98; $p = 0.04$) and RFS (HR 0.52; 95% CI 0.27–1.02; $p = 0.057$) compared with males (Fig. 5A,B).

Population study

Palliative treatment with cisplatin + gemcitabine

For patients undergoing palliative chemotherapy with gemcitabine + cisplatin, median OS was 10.0 months (95% CI 9.6–10.5) in females and 9.8 months (95% CI 9.33–10.20) in males ($p = 0.3$) (Fig. S5).

The HR calculated for 1-year all-cause mortality was 0.97 (95% CI 0.88–1.14, $p = 0.6$) for risk of male sex relative to female sex, suggesting no significant difference in survival for males and females treated with cisplatin + gemcitabine (Table S1).

Adjuvant treatment with single-agent capecitabine

Median OS was 30.7 months (95% CI 27.3–34.1) in females and 26.7 months (95% CI 23.7–29.9) in males ($p = 0.20$). The

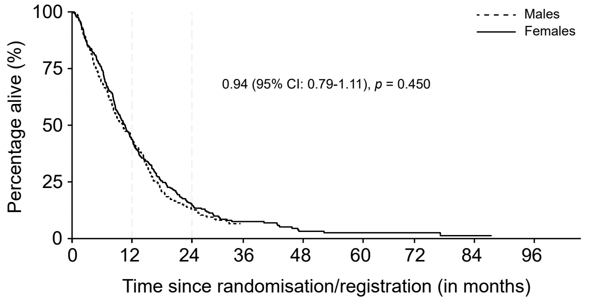

N° at risk																
Males	260	(146)	114	(80)	32	(15)	10	(3)	4	(1)	1	(0)	1	(0)	1	(0)	0
Females	287	(158)	122	(80)	39	(17)	13	(7)	5	(1)	3	(0)	2	(1)	1	(0)	0

Fig. 2. Overall survival curve for ABC studies.

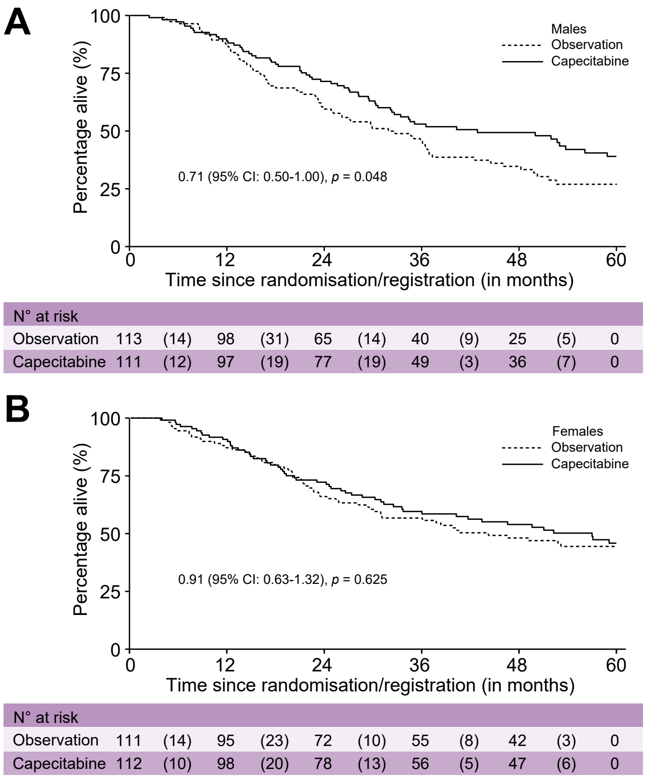

A

N° at risk										
Observation	113	(14)	98	(31)	65	(14)	40	(9)	25	(5)	0
Capecitabine	111	(12)	97	(19)	77	(19)	49	(3)	36	(7)	0

B

N° at risk										
Observation	111	(14)	95	(23)	72	(10)	55	(8)	42	(3)	0
Capecitabine	112	(10)	98	(20)	78	(13)	56	(5)	47	(6)	0

Fig. 3. Overall survival for the BILCAP study comparing 5-year overall survival between observation and capecitabine treatment arms.

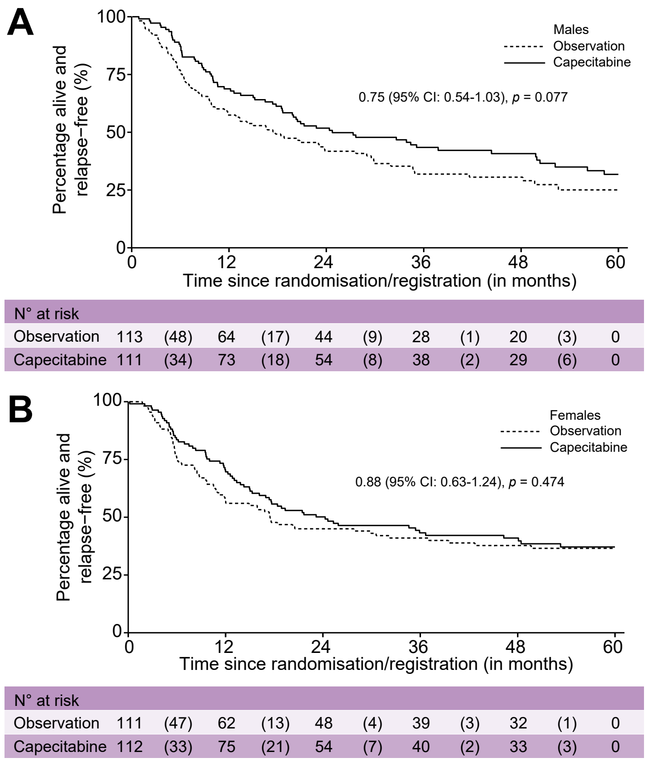

A

N° at risk											
Observation	113	(48)	64	(17)	44	(9)	28	(1)	20	(3)	0
Capecitabine	111	(34)	73	(18)	54	(8)	38	(2)	29	(6)	0

B

N° at risk											
Observation	111	(47)	62	(13)	48	(4)	39	(3)	32	(1)	0
Capecitabine	112	(33)	75	(21)	54	(7)	40	(2)	33	(3)	0

Fig. 4. Relapse-free survival for the BILCAP study comparing 5-year overall survival between observation and capecitabine treatment arms.

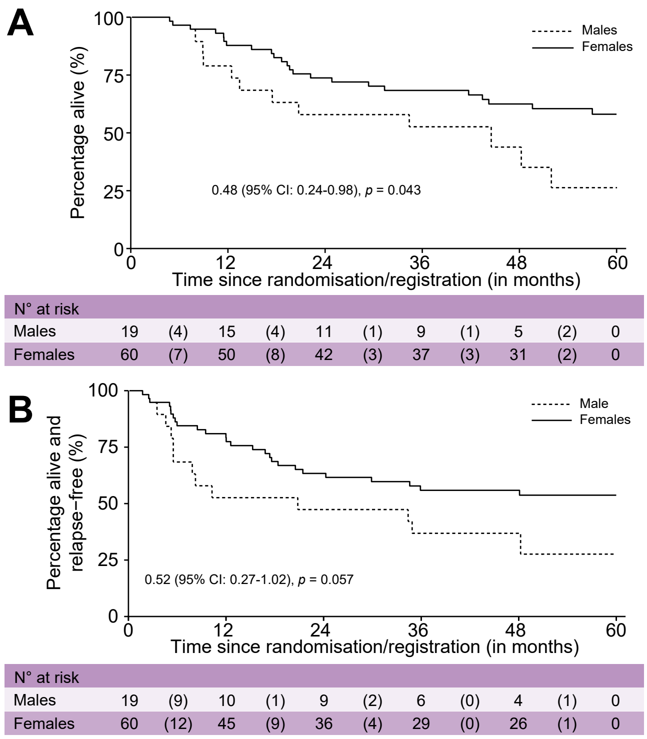

A

N° at risk											
Males	19	(4)	15	(4)	11	(1)	9	(1)	5	(2)	0
Females	60	(7)	50	(8)	42	(3)	37	(3)	31	(2)	0

B

N° at risk											
Males	19	(9)	10	(1)	9	(2)	6	(0)	4	(1)	0
Females	60	(12)	45	(9)	36	(4)	29	(0)	26	(1)	0

Fig. 5. Five-year overall survival for BILCAP patients with gallbladder cancer only, stratified by sex.

hazard ratio for death for females relative to males was 0.85 (95% CI 0.57–1.26; p = 0.40) (Fig. S6, Table S3).

Among patients with GBC, median OS was 33.8 months in females and 26.5 months in males (p = 0.07; n = 103 females, n = 49 males) (Fig. S7). When patients with GBC treated with capecitabine were excluded from the population data, OS remained comparable between sexes. Median OS in this subgroup was 29.1 months (95% CI 25.0–32.0) in females and 26.4 months (95% CI 23.6–30.3) in males (p = 0.81; n = 199 females, n = 238 males).

Discussion

Our analysis of individual patient data from 994 patients in the prospective UK BTC portfolio of studies and 3,953 patients from population data revealed clinically relevant sex differences in AE prevalence, and potential differences in survival benefit from adjuvant chemotherapy between male and female patients treated for BTC.

With regard to AEs, the analysis of gemcitabine-based chemotherapy in the ABC-trials confirms the findings of several studies in other tumour types,[21–24] with other chemotherapies, targeted therapies and immunotherapy[20] that reported substantially higher rates of AEs in females. In females receiving standard-of-care palliative chemotherapy for BTC (cisplatin 25 mg/m^2 and gemcitabine 1,000 mg/m^2 on days 1 and 8 every 3 weeks), this study observed a clinically relevant, nearly twofold increase in several grade ≥3 clinical adverse events: rates of nausea were significantly higher in females, while increased rates of vomiting and fatigue approached statistical significance. These toxicities are most likely related to cisplatin, for which emesis is a particular issue,[31] whereas gemcitabine has relatively low emetogenic potential. Sex differences in the mechanisms of cisplatin-associated toxicity have been described previously (for review, see Marcu 2022[32]) in preclinical studies, with cisplatin-associated toxicity being dependent not only on dose and schedule,[33] but also sex and age. Furthermore, significantly higher rates of non-haematological toxicities, such as grade ≥3 nausea (33% *vs.* 20%, p <0.01) and vomiting (30 *vs.* 18%, p <0.01) have been reported previously in females with lung cancer treated with platinum-based combination chemotherapy.[34] Nephrotoxicity, another significant side effect of cisplatin, occurs at higher rates in peri-menopausal females.[35] By contrast, in children, the risk of cisplatin-induced hearing loss was significantly higher in males (OR 4.812, p = 0.005),[36] confirming that both sex and age modulate the risk of chemotherapy-related AEs. The reason for the overall low rates of nephrotoxicity and ototoxicity in the present study are probably the relatively low cisplatin doses. It is notable that some laboratory AEs, especially thrombocytopenia (146, 56.2% *vs.* 125, 43.6%; p <0.01) were reported more frequently in males, while females showed a higher incidence of clinical AEs such as nausea (213, 74.2% *vs.* 146, 56.2%; p <0.01) and vomiting (150, 52.3% *vs.* 92, 35.4%; p <0.01). This observation could be explained by different mechanisms responsible for and sensitivity to these types of toxicities in male and female patients, which vary according to the type of drug. However, importantly a lower rate of laboratory AEs in females has not been observed in other trials: among patients undergoing adjuvant chemotherapy for colon cancer, the significantly greater rates of haematological

toxicities (leukopenia and neutropenia) in females are associated with higher rates of nausea and vomiting.[37] The higher incidence of specific AEs (nausea, vomiting, fatigue) in women observed in the ABC studies could also be associated with lower incidence of other AEs such as thrombocytopaenia, as dose reductions and treatment delays are frequently used to manage toxicity. This aspect of chemotherapy scheduling may also have influenced the disparity in AEs between males and females observed in these clinical trials.

Our data suggest a potential sex-specific pattern in toxicity from gemcitabine–cisplatin chemotherapy, with higher rates of chemotherapy-induced nausea and vomiting in females and a greater incidence of haematological toxicities in males. Consequently, biologically optimal doses of gemcitabine and cisplatin may differ between the sexes. Furthermore, sex-specific supportive care strategies that account for differential toxicity risks should be considered and may help reduce AEs. In contrast, higher rates of nausea, vomiting and fatigue observed in females treated with capecitabine in the BILCAP-study are in line with previous observations[24] and possibly explained by potentially higher plasma levels of capecitabine and/or its metabolites.[38]

Concerning treatment efficacy, in the BILCAP observation group, there was potentially survival in females compared to males (median survival 44.2 [95% CI not calculated] *vs.* 31.9 [95% CI 23.9–36.9] months); however, this did not reach statistical significance. In the population-based cohort, similar findings were observed, with a point estimate for median OS approximately 4 months lower in males than in females; however, the overlapping 95% CIs preclude a definitive conclusion regarding a statistically significant survival difference among patients treated with adjuvant capecitabine in routine care. Of note, the population study included only patients treated with capecitabine, without a control group, as the data available did not provide data for patients who received no treatment. Thus, these data did not allow for the evaluation of the benefit of adjuvant capecitabine therapy for women treated for BTC. Other studies have reported significantly improved survival in females with early-stage GBC in Japan,[39] which was not observed in the US.[40] As such, an interaction between sex and the biology of GBC is not only plausible, but likely. This finding is consistent with the association between reproductive factors and the risk of GBC,[10] but could not be statistically confirmed in this study and should be prospectively evaluated in a prospective study.

In BILCAP, the benefit of chemotherapy in terms of OS is more pronounced in males, possibly attributable to a difference in disease biology. In males, adjuvant chemotherapy with capecitabine improved survival (HR 0.71, 95% CI 0.50–1.99, p = 0.048), whereas the benefit of capecitabine treatment in females was smaller and did not reach statistical significance (HR 0.91, 95% CI 0.63–1.32, p = 0.625). Thus, considering both the apparent increased toxicity and lesser effectiveness of adjuvant capecitabine in BILCAP, our findings suggest the need for a thorough re-evaluation of the treatment's advantages and risks, as well as the evaluation of other treatment options specifically in female patients with this disease. This finding should be evaluated as well in the ACTICCA-01 study that recently completed accrual, for which the control arm was capecitabine,[41] although this trial did not include an observation-only arm; therefore, the benefit of adjuvant capecitabine for females with BTC should be questioned in future trials.

Importantly, sex differences in cancer biology[13,42] are not limited to BTC. They have been observed in multiple tumour types, with lung cancer being a prime example (for review see Mederos, 2020[43]). These findings support the further investigation of potential sex variations in tumour biology, which should be considered in the design and analysis of future clinical trials to provide an accurate assessment of treatment benefits and risks, especially for patients with GBC.

Our findings should be considered in the context of several limitations. Most important are the limited size of the study population in our trial datasets, the unplanned, *post hoc* nature of the analysis, and the lack of statistical significance of some findings, particularly in relation to efficacy and survival outcomes. Furthermore, we are limited by the lack of pharmacokinetic data. A recent systematic review analysing sex differences in pharmacokinetics, pharmacodynamics and AEs[44] found that differences in pharmacokinetics are strongly predictive of adverse drug reactions for females but not males, highlighting that these differences were not explained only by differences in body weight. Finally, while the use of combination regimens in the ABC-trials makes the interpretation more challenging, this represents a common and clinically relevant situation. A major strength of our study is that it is the first of its kind to provide granular evidence from trial data on survival outcomes, and to compare these outcomes to an independent set of population data. Population data alone would not enable us to determine the types or severity of toxicity; however, this large and diverse dataset confirms the prevalence of our observation of clinically relevant sex differences in AEs, and potentially efficacy, for patients with localized and locally advanced GBC in a real-world setting.

In conclusion, our combined analysis of individual patient data from randomized controlled trials and real-world data suggests clinically relevant sex differences in AEs in the trial setting, with potential survival differences between males and females, although this analysis was limited by sample size and lack of an observational study arm. Differences in AE profiles between male and female patients undergoing standard-of-care, palliative chemotherapy for metastatic biliary cancer were observed, with females being at higher risk. This study adds to the growing body of evidence supporting sex-related differences in cancer biology and treatment outcomes, underscoring the need to further investigate these effects and to incorporate sex as a key variable in the design, analysis, and interpretation of future clinical trials, as well as in the development of tailored treatment and supportive care strategies for male and female patients.

Affiliations

[1]*Department of Oncology, Lausanne University Hospital and University of Lausanne, Switzerland;* [2]*University College London and CRUK Clinical Trials Centre, London, UK;* [3]*UCL School of Pharmacy, London UK;* [4]*University of Manchester / The Christie, Manchester, UK;* [5]*University of Southampton, Department of Surgery, UK;* [6]*University of Leeds and Leeds Teaching Hospitals NHS Trust, Leeds, UK;* [7]*Nuffield Department of Population Health, University of Oxford, Oxford, UK;* [8]*UCL Cancer Institute, London, UK*

Abbreviations

AE, adverse event; BTC, biliary tract cancer; CisGem, cisplatin plus gemcitabine; eCCA, extrahepatic cholangiocarcinoma; GBC, gallbladder cancer; HR, hazard ratio; iCCA, intrahepatic cholangiocarcinoma; NCRAS, National Cancer Registry and Analysis Service; OR, odds ratio; OS, overall survival; PFS, progression-free survival; RFS, recurrence-free survival; SACT, systemic anti-cancer therapy.

Financial support

This work received no funding. JB is partly funded by the UCLH/UCL Biomedical Research Centre. CJT is supported by the NIHR Leeds Clinical Research Facility.

Conflicts of interest

Professor Bridgewater reports grants from MSD and Bristol Myers Squibb outside the submitted work; Anna Wagner reports grants from Bristol Myers Squibb outside the submitted work; Pinkie Chambers reports research grants from Janssen, Pfizer, Tessaro, and Bristol Myers Squibb and Gilead; outside the submitted work.
Please refer to the accompanying ICMJE disclosure forms for further details.

Authors' contributions

AW, JB and PC conceived of the presented idea. All authors contributed to the development of the protocol. AL and LS conducted all analyses. All authors contributed to interpretation of findings. AW, PC and LS produced the first draft of the manuscript. All authors edited this manuscript and approved the final version.

Data availability

The datasets generated during and analysed during the current study are available from the corresponding author on reasonable request.

Previous presentation

ASCO GI Cancer Symposium 2020 (abstract 517).

Supplementary data

Supplementary data to this article can be found online at https://doi.org/10.1016/j.jhepr.2026.101777.

References

1 Mauvais-Jarvis F, Bairey Merz N, Barnes PJ, et al. Sex and gender: modifiers of health, disease, and medicine. Lancet Aug 22 2020;396(10250):565–582. https://doi.org/10.1016/S0140-6736(20)31561-0.
2 Radkiewicz C, Johansson ALV, Dickman PW, Lambe M, Edgren G. Sex differences in cancer risk and survival: a Swedish cohort study. Eur J Cancer Oct 2017;84:130–140. https://doi.org/10.1016/j.ejca.2017.07.013.
3 Dong M, Cioffi G, Wang J, et al. Sex differences in cancer incidence and survival: a pan-cancer analysis. Cancer Epidemiol Biomarkers Prev Jul 2020;29(7):1389–1397. https://doi.org/10.1158/1055-9965.EPI-20-0036.
4 Banales JM, Marin JJG, Lamarca A, et al. Cholangiocarcinoma 2020: the next horizon in mechanisms and management. Nat Rev Gastroenterol Hepatol 2020/09/01 2020;17(9):557–588. https://doi.org/10.1038/s41575-020-0310-z.
5 Mosadeghi S, Liu B, Bhuket T, Wong RJ. Sex-specific and race/ethnicity-specific disparities in cholangiocarcinoma incidence and prevalence in the USA: an updated analysis of the 2000-2011 Surveillance, Epidemiology and End Results registry. Hepatol Res Jun 2016;46(7):669–677. https://doi.org/10.1111/hepr.12605.
6 Jaruvongvanich V, Yang JD, Peeraphatdit T, Roberts LR. The incidence rates and survival of gallbladder cancer in the USA. Eur J Cancer Prev Jan 2019;28(1):1–9. https://doi.org/10.1097/CEJ.0000000000000402.
7 Kim D, Konyn P, Cholankeril G, Bonham CA, Ahmed A. Trends in the mortality of biliary tract cancers based on their anatomical site in the United States from 2009 to 2018. Am J Gastroenterol May 1 2021;116(5):1053–1062. https://doi.org/10.14309/ajg.0000000000001151.
8 Ellington TD, Momin B, Wilson RJ, Henley SJ, Wu M, Ryerson AB. Incidence and mortality of cancers of the biliary tract, gallbladder, and liver by sex, age, race/ethnicity, and stage at diagnosis: United States, 2013 to 2017. Cancer Epidemiol Biomarkers Prev Sep 2021;30(9):1607–1614. https://doi.org/10.1158/1055-9965.EPI-21-0265.
9 Randi G, Malvezzi M, Levi F, et al. Epidemiology of biliary tract cancers: an update. Ann Oncol Jan 2009;20(1):146–159. https://doi.org/10.1093/annonc/mdn533.
10 Jackson SS, Adami HO, Andreotti G, et al. Associations between reproductive factors and biliary tract cancers in women from the Biliary Tract Cancers Pooling Project. J Hepatol Oct 2020;73(4):863–872. https://doi.org/10.1016/j.jhep.2020.04.046.
11 Clocchiatti A, Cora E, Zhang Y, Dotto GP. Sexual dimorphism in cancer. Nat Rev Cancer May 2016;16(5):330–339. https://doi.org/10.1038/nrc.2016.30.
12 Rubin JB, Lagas JS, Broestl L, et al. Sex differences in cancer mechanisms. Biol Sex Differ Apr 15 2020;11(1):17. https://doi.org/10.1186/s13293-020-00291-x.
13 Yuan Y, Liu L, Chen H, et al. Comprehensive characterization of molecular differences in cancer between male and female patients. Cancer Cell May 9 2016;29(5):711–722. https://doi.org/10.1016/j.ccell.2016.04.001.
14 Haupt S, Caramia F, Klein SL, Rubin JB, Haupt Y. Sex disparities matter in cancer development and therapy. Nat Rev Cancer Jun 2021;21(6):393–407. https://doi.org/10.1038/s41568-021-00348-y.
15 Kim HW, Kim JH, Lim BJ, et al. Sex disparity in gastric cancer: female sex is a poor prognostic factor for advanced gastric cancer. Ann Surg Oncol Dec 2016;23(13):4344–4351. https://doi.org/10.1245/s10434-016-5448-0.
16 Schmuck R, Gerken M, Teegen EM, et al. Gender comparison of clinical, histopathological, therapeutic and outcome factors in 185,967 colon cancer patients. Langenbecks Arch Surg Feb 2020;405(1):71–80. https://doi.org/10.1007/s00423-019-01850-6.
17 Keum N, Giovannucci E. Global burden of colorectal cancer: emerging trends, risk factors and prevention strategies. Nat Rev Gastroenterol Hepatol Dec 2019;16(12):713–732. https://doi.org/10.1038/s41575-019-0189-8.
18 Abancens M, Bustos V, Harvey H, McBryan J, Harvey BJ. Sexual dimorphism in colon cancer. Front Oncol 2020;10:607909. https://doi.org/10.3389/fonc.2020.607909.
19 Mauvais-Jarvis F, Berthold HK, Campesi I, et al. Sex- and gender-based pharmacological response to drugs. Pharmacol Rev Apr 2021;73(2):730–762. https://doi.org/10.1124/pharmrev.120.000206.
20 Unger JM, Vaidya R, Albain KS, et al. Sex differences in risk of severe adverse events in patients receiving immunotherapy, targeted therapy, or chemotherapy in cancer clinical trials. J Clin Oncol Feb 4 2022:JCO2102377. https://doi.org/10.1200/JCO.21.02377.
21 Singh S, Parulekar W, Murray N, et al. Influence of sex on toxicity and treatment outcome in small-cell lung cancer. J Clin Oncol Feb 1 2005;23(4):850–856. https://doi.org/10.1200/JCO.2005.03.171.
22 Klimm B, Engert A. Differences in hematotoxicity between male and female patients with Hodgkin lymphoma and other malignancies. Nat Clin Pract Oncol Jun 2008;5(6):316–323. https://doi.org/10.1038/ncponc1124.
23 van den Berg H, Paulussen M, Le Teuff G, et al. Impact of gender on efficacy and acute toxicity of alkylating agent -based chemotherapy in Ewing sarcoma: secondary analysis of the Euro-Ewing99-R1 trial. Eur J Cancer Nov 2015;51(16):2453–2464. https://doi.org/10.1016/j.ejca.2015.06.123.
24 Wagner AD, Grothey A, Andre T, et al. Sex and adverse events of adjuvant chemotherapy in colon cancer: an analysis of 34,640 patients in the ACCENT database. J Natl Cancer Inst Aug 24 2020. https://doi.org/10.1093/jnci/djaa124.
25 Primrose JN, Fox RP, Palmer DH, et al. Capecitabine compared with observation in resected biliary tract cancer (BILCAP): a randomised, controlled, multicentre, phase 3 study. Lancet Oncol May 2019;20(5):663–673. https://doi.org/10.1016/S1470-2045(18)30915-X.
26 Valle J, Wasan H, Palmer DH, et al. Cisplatin plus gemcitabine *vs.* gemcitabine for biliary tract cancer. N Engl J Med Apr 8 2010;362(14):1273–1281. https://doi.org/10.1056/NEJMoa0908721.
27 Bridgewater J, Lopes A, Beare S, et al. A phase 1b study of Selumetinib in combination with Cisplatin and Gemcitabine in advanced or metastatic biliary tract cancer: the ABC-04 study. BMC Cancer Feb 24 2016;16:153. https://doi.org/10.1186/s12885-016-2174-8.
28 Valle JW, Wasan H, Lopes A, et al. Cediranib or placebo in combination with cisplatin and gemcitabine chemotherapy for patients with advanced biliary tract cancer (ABC-03): a randomised phase 2 trial. Lancet Oncol Aug 2015;16(8):967–978. https://doi.org/10.1016/S1470-2045(15)00139-4.
29 Bright CJ, Lawton S, Benson S, et al. Data Resource profile: the systemic anti-cancer therapy (SACT) dataset. Int J Epidemiol Feb 1 2020;49(1). https://doi.org/10.1093/ije/dyz137. 15-15l.
30 Henson KE, Elliss-Brookes L, Coupland VH, et al. Data Resource profile: national cancer registration dataset in England. Int J Epidemiol 2019;49(1). https://doi.org/10.1093/ije/dyz076. 16-16h.
31 Go RS, Adjei AA. Review of the comparative pharmacology and clinical activity of cisplatin and carboplatin. J Clin Oncol Jan 1999;17(1):409–422. https://doi.org/10.1200/JCO.1999.17.1.409.

32 Marcu LG. Gender and sex-related differences in normal tissue effects induced by platinum compounds. Pharmaceuticals (Basel) Feb 20 2022;15(2). https://doi.org/10.3390/ph15020255.
33 Jilanchi S, Talebi A, Nematbakhsh M. Cisplatin alters sodium excretion and renal clearance in rats: gender and drug dose related. Adv Biomed Res 2018;7:54. https://doi.org/10.4103/abr.abr_124_17.
34 Wakelee HA, Wang W, Schiller JH, et al. Survival differences by sex for patients with advanced non-small cell lung cancer on Eastern Cooperative Oncology Group trial 1594. J Thorac Oncol Jun 2006;1(5):441–446.
35 Chen WY, Hsiao CH, Chen YC, et al. Cisplatin nephrotoxicity might have a sex difference. An analysis based on women's sex hormone changes. J Cancer 2017;8(19):3939–3944. https://doi.org/10.7150/jca.20083.
36 Yancey A, Harris MS, Egbelakin A, Gilbert J, Pisoni DB, Renbarger J. Risk factors for cisplatin-associated ototoxicity in pediatric oncology patients. Pediatr Blood Cancer Jul 15 2012;59(1):144–148. https://doi.org/10.1002/pbc.24138.
37 Wagner AD, Grothey A, Andre T, et al. Sex and adverse events of adjuvant chemotherapy in colon cancer: an analysis of 34 640 patients in the ACCENT database. J Natl Cancer Inst Apr 6 2021;113(4):400–407. https://doi.org/10.1093/jnci/djaa124.
38 Chachad S, Purandare S, Malhotra G, Naidu R. Comparison of pharmacokinetics and safety profiles of two capecitabine tablet formulations in patients with colon, colorectal or breast cancer. Cancer Chemother Pharmacol Feb 2013;71(2):287–292. https://doi.org/10.1007/s00280-012-2007-7.
39 Kayahara M, Nagakawa T. Recent trends of gallbladder cancer in Japan: an analysis of 4,770 patients. Cancer Aug 1 2007;110(3):572–580. https://doi.org/10.1002/cncr.22815.
40 Rawla P, Sunkara T, Thandra KC, Barsouk A. Epidemiology of gallbladder cancer. Clin Exp Hepatol May 2019;5(2):93–102. https://doi.org/10.5114/ceh.2019.85166.
41 Stein A, Arnold D, Bridgewater J, et al. Adjuvant chemotherapy with gemcitabine and cisplatin compared to observation after curative intent resection of cholangiocarcinoma and muscle invasive gallbladder carcinoma (ACTICCA-1 trial) - a randomized, multidisciplinary, multinational phase III trial. BMC Cancer 2015;15(1):564. https://doi.org/10.1186/s12885-015-1498-0.
42 Li CH, Prokopec SD, Sun RX, et al. Sex differences in oncogenic mutational processes. Nat Commun Aug 28 2020;11(1):4330. https://doi.org/10.1038/s41467-020-17359-2.
43 Mederos N, Friedlaender A, Peters S, Addeo A. Gender-specific aspects of epidemiology, molecular genetics and outcome: lung cancer. ESMO Open Nov 2020;5(Suppl 4):e000796. https://doi.org/10.1136/esmoopen-2020-000796.
44 Zucker I, Prendergast BJ. Sex differences in pharmacokinetics predict adverse drug reactions in women. Biol Sex Differ Jun 5 2020;11(1):32. https://doi.org/10.1186/s13293-020-00308-5.

Keywords: biological sex; systemic anti-cancer therapy; treatment response; Biliary tract cancer; toxicity; Survival.

Received 21 February 2025; received in revised form 29 January 2026; accepted 30 January 2026; Available online 20 February 2026

Journal of Hepatology, Volume *8*

Supplemental information

Impact of sex on chemotherapy toxicity and efficacy in biliary tract cancer: Analysis of ABC, BILCAP trials and population data

Anna D. Wagner, Andre Lopes, Pinkie Chambers, Juan W. Valle, John Primrose, Chris Twelves, Luke Steventon, Zhe Wang, David Dodwell, and John Bridgewater

Impact of sex on chemotherapy toxicity and efficacy in biliary tract cancer: Analysis of ABC, BILCAP trials and population data

Anna D. Wagner, Andre Lopes, Pinkie Chambers, Juan W. Valle, John Primrose, Chris Twelves, Luke Steventon, Zhe Wang, David Dodwell, John Bridgewater

Table of contents

Expanded Methodology – Population Study……………………………………………...…2

Fig. S1……………………………………………………………………………………...…3

Fig. S2……………………………………………………………………………………...…5

Fig. S3……………………………………………………………………………………...…6

Fig. S4……………………………………………………………………………………...…7

Fig. S5……………………………………………………………………………………...…8

Fig. S6……………………………………………………………………………………...…9

Fig. S7…………………………………………………………………………………….....10

Table S1…………………………………………………………………………………….. 11

Table S2……………………………………………………………………………………..12

Table S3……………………………………………………………………………………..14

Expanded Methodology - Population Study

Methods

The National Cancer Registry Database (NCRD) records diagnoses of all cancer patients treated within the NHS or NHS-funded centres in England. This registry provides a comprehensive record of >99% of patients with cancer in England. Cancer diagnosis is recorded using standardised ICD-10 codes. The Systemic Anti-Cancer Therapy (SACT) Dataset provided systemic treatment information that was linked to the NCRD patient records.

Inclusion and exclusion criteria

Patients with a diagnosis of biliary tract cancers were identified using ICD-10 codes (BTC, C22, extrahepatic cancer, C23, gallbladder cancer, C24, extrahepatic bile duct cancer).

Patients receiving cisplatin + gemcitabine combination or capecitabine monotherapy as first-line therapy were identified algorithmically using drug administration data available in the Systemic Anti-Cancer Therapy (SACT) Dataset.

Statistical analysis

Kaplan-Meier estimator was used to compare 1 and 5 year all-cause mortality between male and female treatment groups for each SACT protocol.

Cox regression analysis was performed for BTC patients treated with cisplatin + gemcitabine, or capecitabine monotherapy respectively as first-line systemic therapy. 1 year and 5 year all-cause mortality time was calculated as time from first chemotherapy treatment to death by any cause.

Results:

7,196 patients with a diagnosis of BTC were identified in the NCRD registry. Of these, 5,038 patients had a corresponding record of systemic treatment in the SACT dataset.

Of 5,565 patients who received SACT for BTC, 3,362 received cisplatin + gemcitabine in the first-line setting and 591 received capecitabine monotherapy.

Median age was 66 (IQR 58-72) overall. The study period was 02/04/2012 (first patient initiating chemotherapy) to 16/06/2022 (last patient initiating chemotherapy).

Fig. S1A: Patient disposition across trials and analysis subgroups

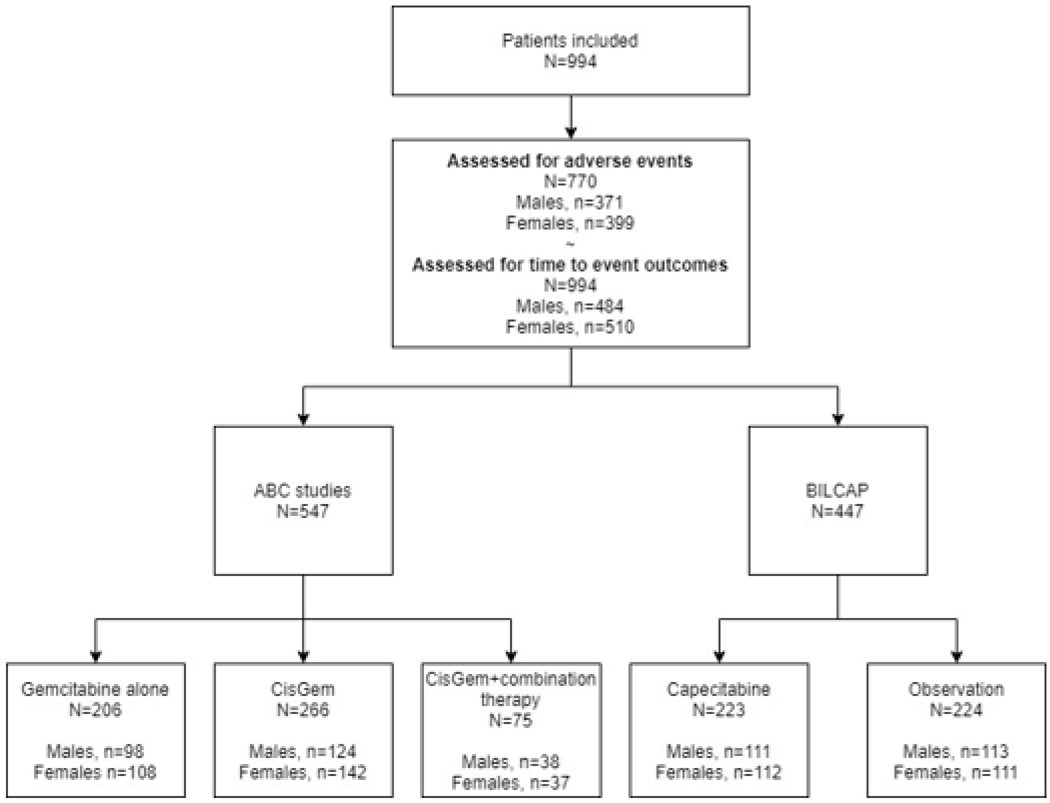

Fig. S1B: Patients included in population analysis

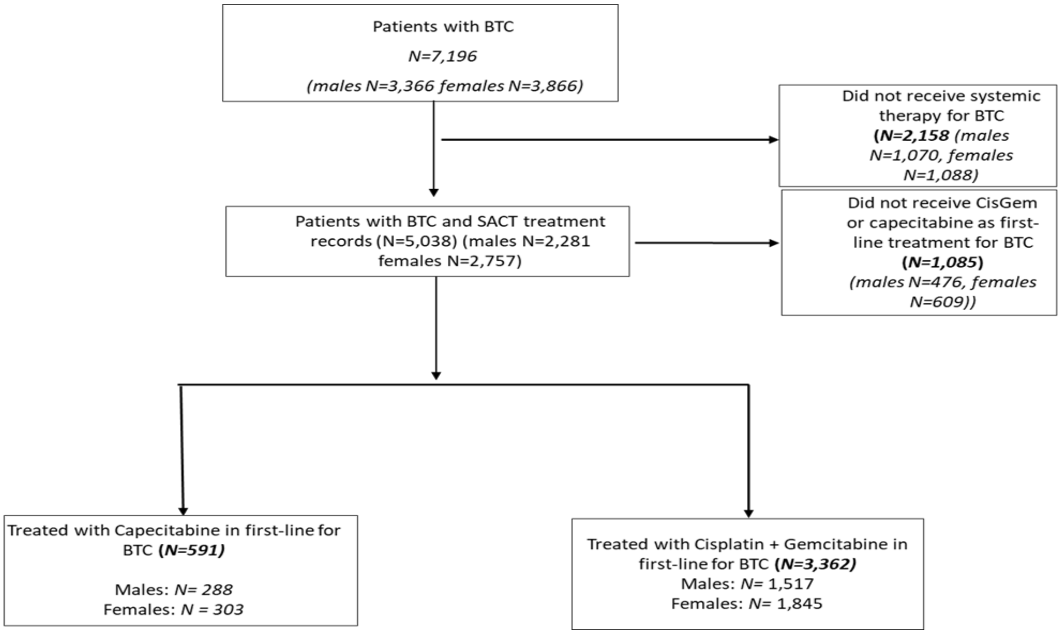

Abbreviations: BTC- biliary tract cancers; SACT- systemic anticancer treatments

Fig. S2: Grade 3-5 adverse events reported in ABC studies in ≥15 patients.

	Males N(%), N=260	Females N(%), N=287	OR(95%CI)	p
General disorders and administration site conditions				
Fatigue	10 (3.8%)	23 (8.0%)	2.18 (1.02;4.67)	0.045
Pain	24 (9.2%)	32 (11.1%)	1.23 (0.71;2.16)	0.460
Gastrointestinal disorders				
Nausea	7 (2.7%)	15 (5.2%)	1.99 (0.80;4.97)	0.139
Vomiting	13 (5.0%)	27 (9.4%)	1.97 (1.00;3.91)	0.052
Ascites	6 (2.3%)	9 (3.1%)	1.37 (0.48;3.90)	0.555
Abdominal pain	8 (3.1%)	11 (3.8%)	1.26 (0.50;3.17)	0.630
Diarrhea	12 (4.6%)	14 (4.9%)	1.06 (0.48;2.34)	0.885
Blood and lymphatic system disorders				
Febrile neutropenia	16 (6.2%)	26 (9.1%)	1.52 (0.80;2.90)	0.205
Anemia	22 (8.5%)	21 (7.3%)	0.85 (0.46;1.59)	0.620
Nervous system disorders				
Lethargy	40 (15.4%)	58 (20.2%)	1.39 (0.89;2.17)	0.143
Investigations				
Aspartate aminotransferase increased	18 (6.9%)	26 (9.1%)	1.34 (0.72;2.50)	0.360
Blood bilirubin increased	28 (10.8%)	35 (12.2%)	1.15 (0.68;1.95)	0.602
Alanine aminotransferase increased	29 (11.2%)	32 (11.1%)	1.00 (0.59;1.70)	0.999
GGT increased	40 (15.4%)	40 (13.9%)	0.89 (0.55;1.43)	0.632
Neutrophil count decreased	70 (26.9%)	68 (23.7%)	0.84 (0.57;1.24)	0.385
Alkaline phosphatase increased	10 (3.8%)	9 (3.1%)	0.81 (0.32;2.02)	0.651
Platelet count decreased	27 (10.4%)	23 (8.0%)	0.75 (0.42;1.35)	0.338
White blood cell decreased	42 (16.2%)	32 (11.1%)	0.65 (0.40;1.07)	0.089
Respiratory, thoracic and mediastinal disorders				
Dyspnea	9 (3.5%)	12 (4.2%)	1.22 (0.50;2.94)	0.662
Vascular disorders				
Thromboembolic event	17 (6.5%)	17 (5.9%)	0.90 (0.45;1.80)	0.766
Hypertension	23 (8.8%)	15 (5.2%)	0.57 (0.29;1.11)	0.100
Metabolism and nutrition disorders				
Anorexia	12 (4.6%)	11 (3.8%)	0.82 (0.36;1.90)	0.649
Hypernatremia/Hyponatremia	8 (3.1%)	7 (2.4%)	0.79 (0.28;2.20)	0.649
Infections and infestations				
Sepsis	9 (3.5%)	8 (2.8%)	0.80 (0.30;2.10)	0.651
Biliary tract infection	23 (8.8%)	18 (6.3%)	0.69 (0.36;1.31)	0.256
Infections and infestations (unspecified)	29 (11.2%)	21 (7.3%)	0.63 (0.35;1.13)	0.122

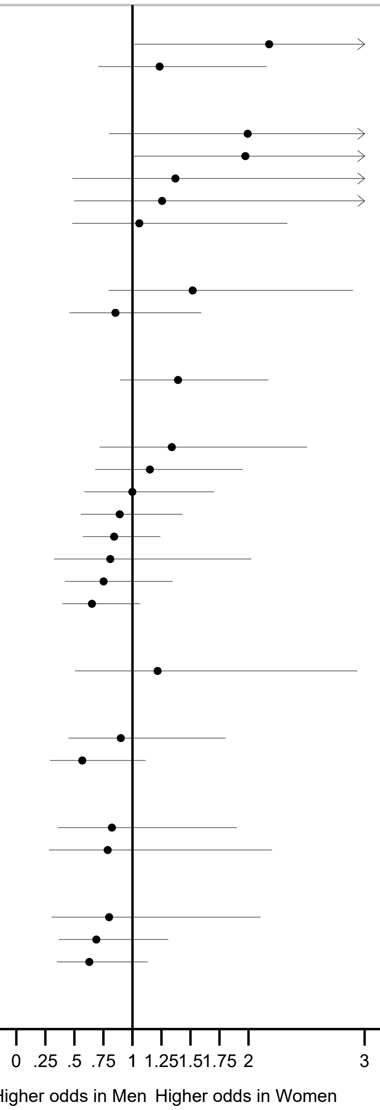

Fig. S2 depicts grade 3-5 AEs in the ABC studies reported in >15 patients. None of these AEs achieved a significance level of 0.01, although this was approached by fatigue (23, 8.0% vs 10, 3.8%; OR: 2.18, 95%CI: 1.02; 4.67, p=0.045) significance. Other relevant reported AEs grade >3 were numerically more frequent in females (58, 20.2% vs 40, 15.4%; OR: 1.39, 95%CI: 0.89; 2.17, p=0.14), vomiting (27, 9.4% vs 13, 5.0%; OR:

1.97, 95%CI: 1.00; 3.91, p=0.05), and nausea (15, 5.2% vs 7, 2.7%; OR: 1.99, 95%CI: 0.80; 4.97, p=0.14). In contrast, trends were reported towards greater rates in males of grade ≥3 leukopenia (42, 16.2% vs 32, 11.1%; OR: 0.65, 95%CI: 0.40; 1.07, p=0.09), hypertension (23, 8.8% vs 15, 5.2%; OR: 0.57, 95%CI: 0.29; 1.11, p=0.10) and infections/infestations (29, 11.2% vs 21, 7.3%; OR: 0.63, 95%CI: 0.35; 1.13, p=0.12).

Fig. S3. Adverse event of any grade (including grade 5) reported in BILCAP in ≥15 patients

CTCAE term 4.03	Males N(%), N=111	Females N(%), N=112	OR(95%CI)	p
Investigations				
Weight loss	11 (9.9%)	18 (16.1%)	1.74 (0.78;3.88)	0.175
Neutrophil count decreased	26 (23.4%)	23 (20.5%)	0.84 (0.45;1.59)	0.603
Platelet count decreased	14 (12.6%)	12 (10.7%)	0.83 (0.37;1.89)	0.659
Investigations (unspecified)	23 (20.7%)	19 (17.0%)	0.78 (0.40;1.53)	0.474
Alkaline phosphatase increased	10 (9.0%)	8 (7.1%)	0.78 (0.29;2.05)	0.610
Blood bilirubin increased	27 (24.3%)	18 (16.1%)	0.60 (0.31;1.16)	0.127
Blood and lymphatic system disorders				
Anemia	47 (42.3%)	37 (33.0%)	0.67 (0.39;1.16)	0.152
Eye disorders				
Eye disorders (unspecified)	13 (11.7%)	5 (4.5%)	0.35 (0.12;1.02)	0.055
Gastrointestinal disorders				
Vomiting	16 (14.4%)	34 (30.4%)	2.59 (1.33;5.03)	0.005
Nausea	42 (37.8%)	66 (58.9%)	2.36 (1.38;4.03)	0.002
Diarrhea	62 (55.9%)	75 (67.0%)	1.60 (0.93;2.76)	0.089
Mucositis oral	45 (40.5%)	56 (50.0%)	1.47 (0.86;2.49)	0.157
Abdominal pain	31 (27.9%)	40 (35.7%)	1.43 (0.81;2.53)	0.213
Constipation	17 (15.3%)	18 (16.1%)	1.06 (0.51;2.18)	0.877
Dyspepsia	11 (9.9%)	8 (7.1%)	0.70 (0.27;1.81)	0.461
General disorders and administration site conditions				
Fatigue	80 (72.1%)	95 (84.8%)	2.17 (1.12;4.20)	0.022
Fever	18 (16.2%)	15 (13.4%)	0.80 (0.38;1.68)	0.553
Injury, poisoning and procedural complications				
Wound complication	5 (4.5%)	10 (8.9%)	2.08 (0.69;6.29)	0.195
Metabolism and nutrition disorders				
Anorexia	11 (9.9%)	17 (15.2%)	1.63 (0.72;3.65)	0.238
Musculoskeletal and connective tissue disorders				
Arthralgia	6 (5.4%)	13 (11.6%)	2.30 (0.84;6.28)	0.105
Back pain	7 (6.3%)	8 (7.1%)	1.14 (0.40;3.27)	0.803
Nervous system disorders				
Dizziness	6 (5.4%)	14 (12.5%)	2.50 (0.92;6.76)	0.071
Dysgeusia	12 (10.8%)	14 (12.5%)	1.18 (0.52;2.68)	0.695
Nervous system disorders (unspecified)	10 (9.0%)	10 (8.9%)	0.99 (0.40;2.48)	0.983
Skin and subcutaneous tissue disorders				
Alopecia	4 (3.6%)	16 (14.3%)	4.46 (1.44;13.80)	0.010
Nail discoloration	4 (3.6%)	11 (9.8%)	2.91 (0.90;9.45)	0.075
Skin and subcutaneous tissue disorders (unspecified)	84 (75.7%)	91 (81.3%)	1.39 (0.73;2.65)	0.312
Pruritus	12 (10.8%)	7 (6.3%)	0.55 (0.21;1.45)	0.228

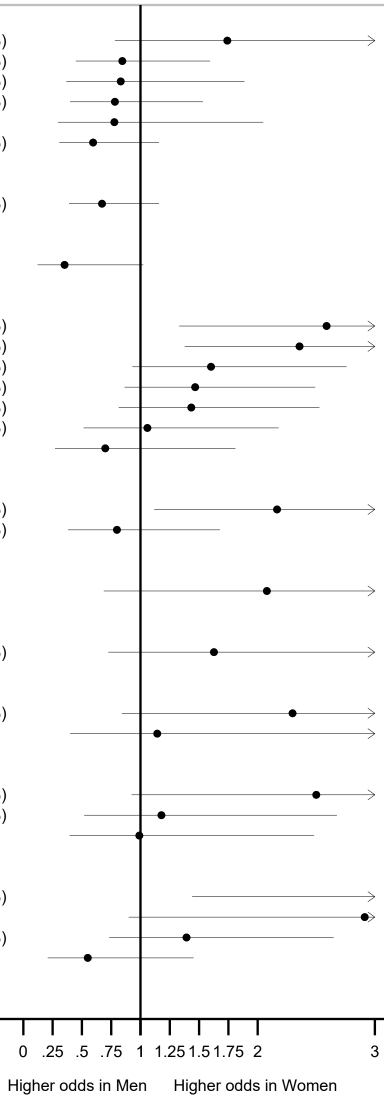

Fig. S3. Laboratory AEs again appeared more common in males, but differences were not statistically significant. Most clinical AEs were numerically greater in females; however, only vomiting (34, 30.4% vs 16, 14.4%, $p \leq 0.01$), nausea (66, 58.9% vs 42, 37.8%, $p \leq 0.01$), and alopecia (16, 14.3% vs 4, 3.6%, p=0.01) achieved statistical significance, while fatigue approached statistical significance (95, 84.8% vs 80, 72.1%, p=0.02).

Fig. S4. Overall survival by sex in BILCAP

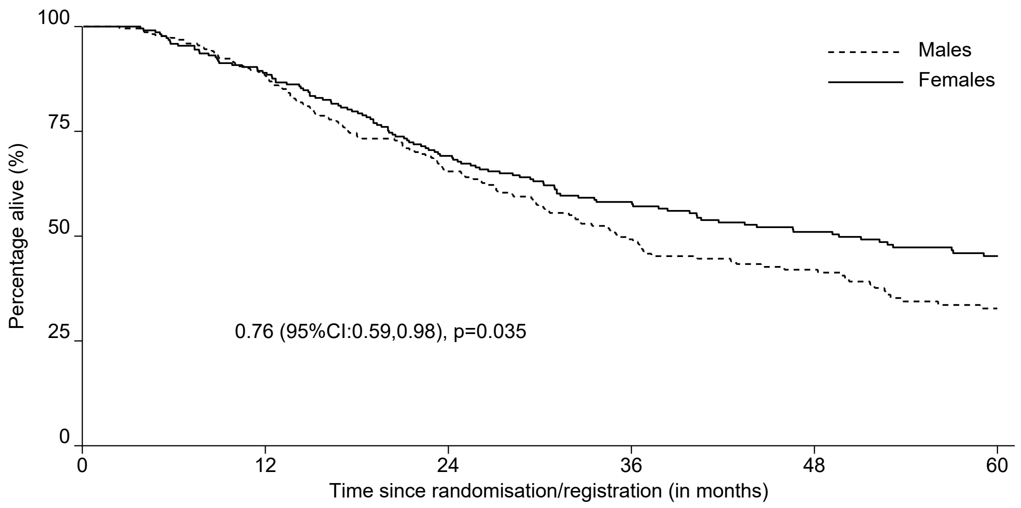

Number at risk

Males	224	(26)	195	(50)	142	(33)	89	(12)	61	(12)	0
Females	223	(24)	193	(43)	150	(23)	111	(13)	89	(9)	0

Fig. S5: 5-year OS by sex in metastatic BTC patients treated with first-line Cisplatin + Gemcitabine as per ABC protocol (population study).

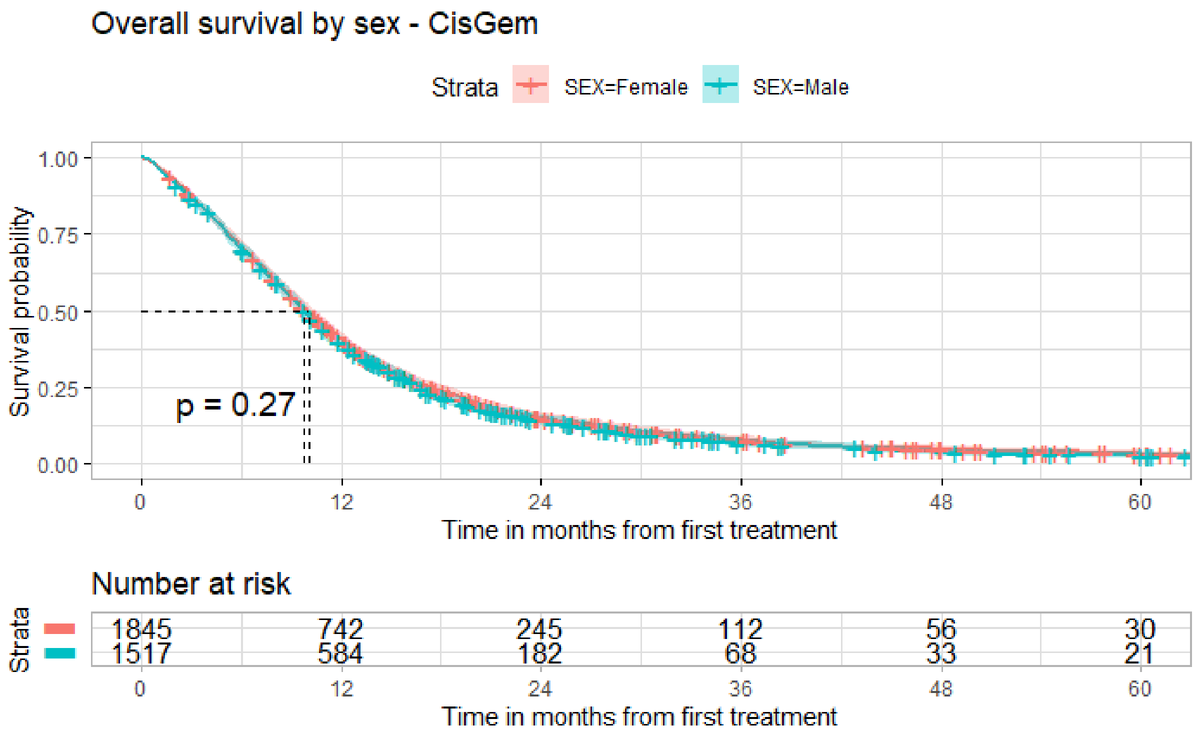

Fig. S6: 5-year OS by sex in patients with localized/ locally advanced BTC patients treated with adjuvant Capecitabine (population study).

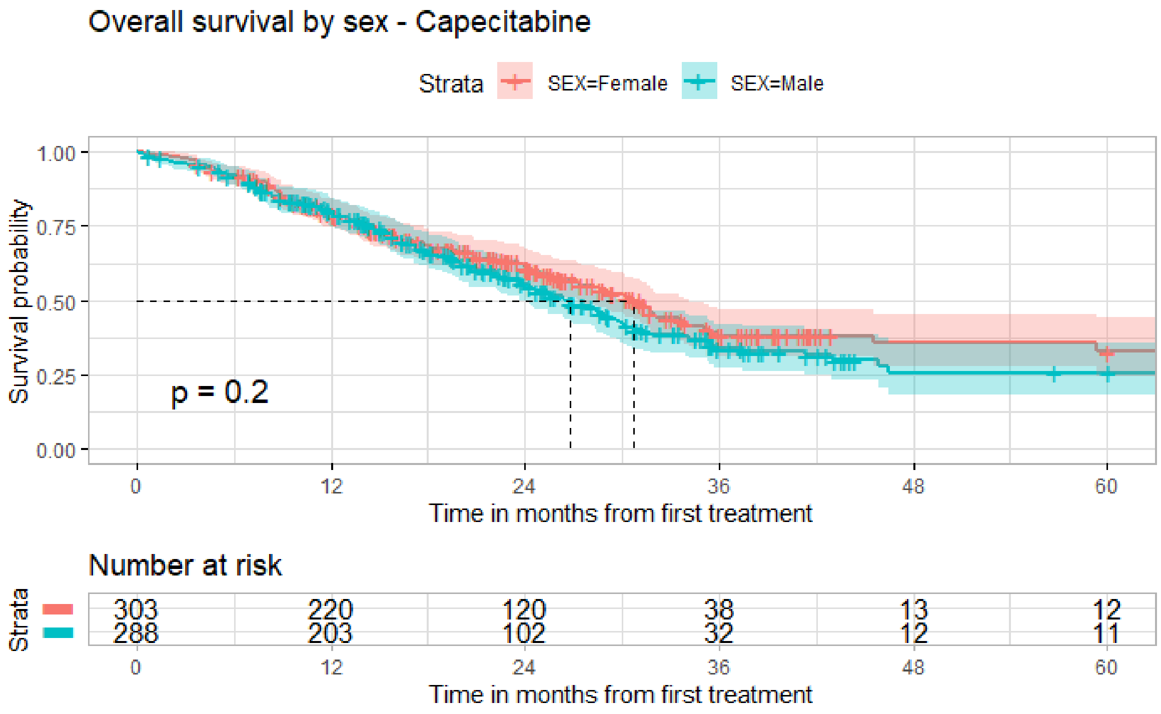

Fig. S7: 5-year OS by sex in gallbladder cancer patients treated with Capecitabine as per BILCAP protocol (population study).

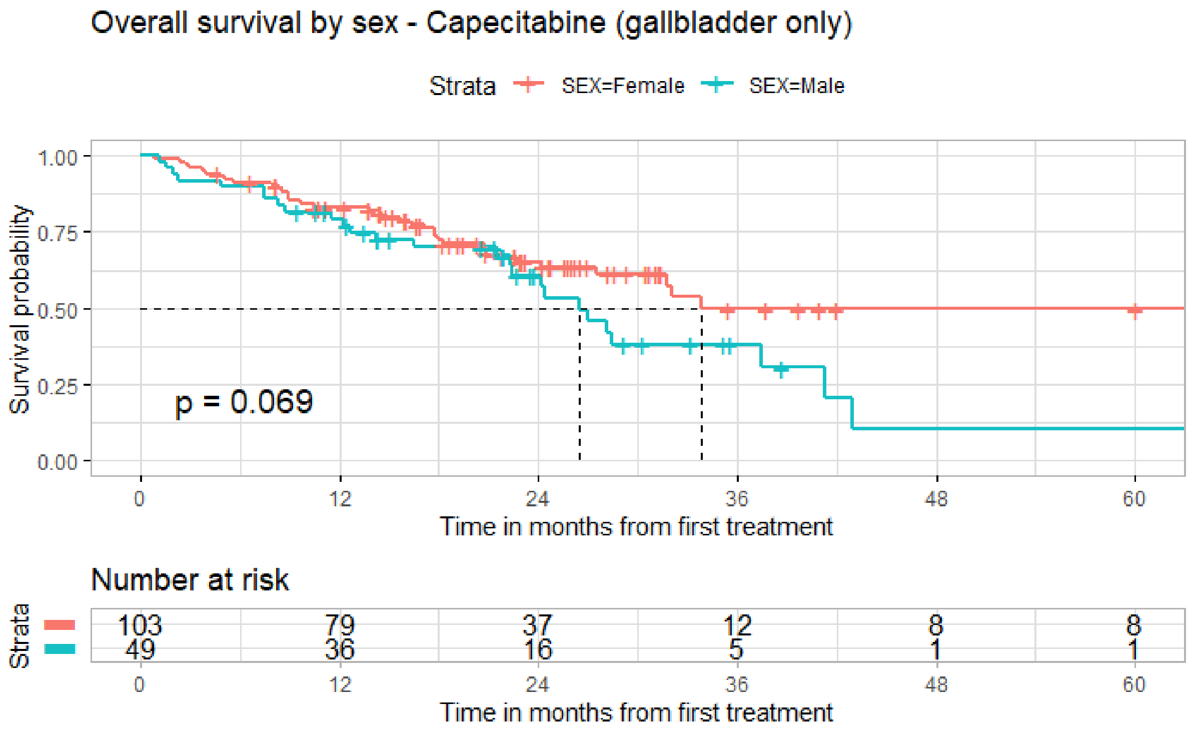

Table S1: Descriptive statistics of patients in national data for England treated with cisplatin + gemcitabine or capecitabine monotherapy

Characteristic	Overall<br>N = 3,953[1]	Cisplatin + Gemcitabine<br>N = 3,362[1]	Capecitabine<br>N = 591[1]
Sex			
Female	2,148 (54%)	1,845 (55%)	303 (51%)
Male	1,805 (46%)	1,517 (45%)	288 (49%)
Age	66 (58, 72)	66 (58, 71)	67 (59, 73)
Diagnosis (ICD-10 code)			
Intrahepatic cholangiocarcinoma (C22.1)	2,120 (53%)	1,883 (56%)	237 (40%)
Cancer of gallbladder (C23.0)	1,059 (27%)	906 (27%)	153 (26%)
Cancer of extrahepatic or unspecified/other cancer of biliary tract (C24.0, C24.8, C24.9)	774 (19%)	573 (17%)	201 (34%)
Stage			
1	127 (3.2%)	72 (2.1%)	55 (9.3%)
2	466 (12%)	275 (8.2%)	191 (32%)
3	499 (13%)	350 (10%)	149 (25%)
4	1,774 (45%)	1,691 (50%)	83 (14%)
Unknown	1,087 (27%)	974 (29%)	113 (19%)
Ethnicity			
Asian	192 (4.9%)	166 (4.9%)	26 (4.4%)
Black	102 (2.6%)	94 (2.8%)	8 (1.4%)
Mixed Race	21 (0.5%)	18 (0.5%)	3 (0.5%)
Other	76 (1.9%)	64 (1.9%)	12 (2.0%)

Characteristic	Overall<br>N = 3,953[1]	Cisplatin + Gemcitabine<br>N = 3,362[1]	Capecitabine<br>N = 591[1]
Unknown	95 (2.3%)	81 (2.3%)	14 (2.4%)
White	3,467 (88%)	2,939 (88%)	528 (89%)

[1] n (%); Median (IQR)

Table S2: Male vs female survival in real-world patients receiving cisplatin + gemcitabine.

Characteristic	HR[1]	95% CI[1]	p-value
Sex			
Female	0.97	0.88, 1.14	0.6
Male	-	-	-
Age	1.01	1.00, 1.01	0.001
Charlson comorbidity index			
0	0.86	0.76, 0.97	0.018
≥1	—	—	
Ethnicity			
Asian	—	—	
Black	1.36	0.93, 1.99	0.11
Mixed Race	1.22	0.61, 2.45	0.6
Other	1.37	0.91, 2.06	0.13
Unknown	2.01	1.40, 2.88	<0.001

Characteristic	HR[1]	95% CI[1]	p-value
White	1.44	1.13, 1.84	0.003
Stage			
1	—	—	
2	1.20	0.78, 1.86	0.4
3	1.26	0.82, 1.92	0.3
4	2.16	1.45, 3.21	<0.001
Unknown	1.58	1.06, 2.37	0.026

[1] HR = Hazard Ratio, CI = Confidence Interval

Table S3: Cox regression analysis of relative risk of male vs female sex in real-world patients receiving single-agent capecitabine.

Characteristic	HR[1]	95% CI[1]	p-value
Sex			
Female	0.85	0.57, 1.26	0.4
Male	-	-	-
Age	1.01	1.0, 1.03	0.2
Ethnicity			
White	—	—	
Asian	0.89	0.28, 2.83	0.8
Black	1.27	0.17, 9.40	0.8
Mixed Race	0.00	0.00, Inf	>0.9
Other	3.08	1.10, 8.60	0.032
Unknown	3.27	1.30, 8.24	0.012
Charlson comorbidity index			
0	—	—	
≥1	1.29	0.80, 2.08	0.3
Stage			
1	—	—	
2	1.00	0.32, 3.15	>0.9
3	2.99	1.04, 8.60	0.043
4	9.93	3.49, 28.3	<0.001
Unknown	4.04	1.38, 11.8	0.011

[1] HR = Hazard Ratio, CI = Confidence Interval
